# Engineered strains as living factories: efficient biosynthesis and modification of natural products via synthetic biology and one-pot chemical tandem reactions

**DOI:** 10.3389/fmicb.2026.1891602

**Published:** 2026-08-18

**Authors:** Sha Liu, Pan Chen, Haitao Li, Wenting Dong, Qiaoqiao Xu

**Affiliations:** 1 School of Chemical Engineering and Pharmacy, Wuhan Institute of Technology, Wuhan, Hubei, China; 2 Wuhan Grand Hoyo Co., Ltd., Wuhan, Hubei, China; 3 Admin Office, Huangshi Public Health Medical Center, Huangshi, Hubei, China; 4 Haisco Pharmaceutical Group Co., Ltd., Chengdu, Sichuan, China; 5 Hubei Institute of Veterinary Drug Control, Wuhan, Hubei, China; 6 Department of Pharmacy, The Fifth Affiliated Hospital, Sun Yat-sen University, Zhuhai, Guangdong, China

**Keywords:** cell-free biomanufacturing, chemoenzymatic synthesis, engineered strains, natural products, one-pot tandem catalysis, synthetic biology

## Abstract

Natural products are a crucial source for drug discovery, but their traditional development is limited by resource availability, efficiency, chemical complexity, and the difficulty of rapidly diversifying biologically active scaffolds. This review addresses the central question of how biosynthetic precision and chemical diversification can be coupled when conventional metabolic engineering alone cannot fully overcome low titers, enzyme promiscuity limits, intermediate toxicity, and scale-up heterogeneity. We examine engineered strains as microbial living factories that construct stereochemically complex cores from renewable feedstocks, and one-pot chemical tandem reactions as complementary tools for post-biosynthetic functionalization, skeletal remodeling, and rapid analog generation. The review compares natural-product classes with distinct bottlenecks, analyzes mass-transfer and oxygen-limitation challenges during industrial translation, and explains domino, multicomponent, and sequential one-pot mechanisms for a microbiology-oriented readership. Recent advances in biocompatible catalysis, chemoenzymatic cascades, artificial metalloenzymes, photobiocatalysis, flow biocatalysis, and cell-free synthetic platforms are discussed as three integration paradigms: post-fermentation modification, one-pot chemoenzymatic coupling, and open cell-free manufacturing. Finally, the review outlines a roadmap for catalyst compatibility, process scale-up, regulatory assessment, and automated design-build-test-learn workflows to promote sustainable natural-product production and lead discovery.

## Graphical abstract

**Figure:**
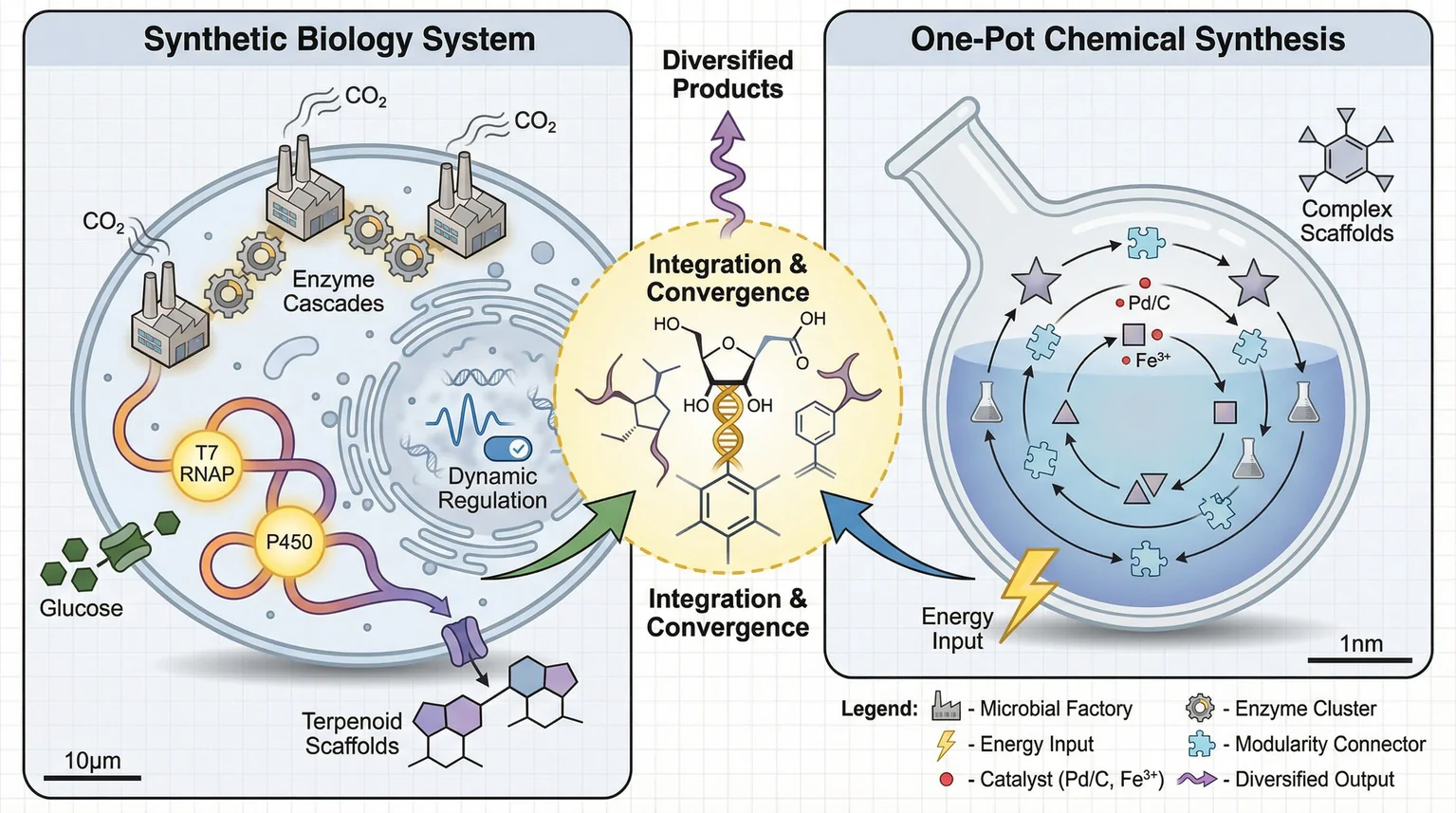

## 1 Introduction

Natural products (NPs) and their derivatives have long been indispensable resources in pharmaceutical development, agriculture, and the chemical industry. Statistics indicate that approximately one-third of small-molecule drugs approved globally over the past four decades are directly derived from natural products and their derivatives (Zhang Y. et al., 2025). Since the landmark discovery of penicillin, NPs have formed the foundation of anti-infectives, immunosuppressants, chemotherapeutic agents, and diverse enzyme inhibitors (Wang et al., 2021). However, traditional development methods relying on direct extraction from natural sources or step-wise chemical synthesis face significant challenges in the 21st century, including structural complexity, low yields, resource depletion, and environmental concerns (Huang et al., 2011; Rice et al., 2025). While pure chemical synthesis can provide defined structures, it often involves lengthy steps, low overall yields, and high costs for molecules with complex chiral centers and polycyclic systems such as erythromycin and paclitaxel, making industrial-scale production difficult (Rice et al., 2025). Furthermore, the emergence of multidrug-resistant pathogens has rendered traditional NPs obsolete, necessitating the exploration of novel NPs with more unique mechanisms of action (Zhang Y. et al., 2025).

To overcome these limitations, synthetic biology has spurred the revolutionary concept of “microbial cell factories,” where microorganisms (e.g., *Escherichia coli*, yeast) are engineered into highly tailored “living factories” that harness their native metabolic networks to heterologously express biosynthetic gene clusters from plants, animals, or microorganisms, thereby enabling efficient, green, and sustainable production of target compounds (Ke and Yoshikuni, 2020). For instance, reconstruction of complex metabolic pathways in *E. coli* and *Saccharomyces cerevisiae* has allowed the biosynthesis of plant-derived terpenoids, bacterial polyketides, and non-natural derivatives with enhanced bioactivity (Trinh and Mendoza, 2016). Remarkably, Wang et al. (2025) achieved a record titer of 124.3 g/L for d-pantothenic acid through systematic remodeling of *E. coli* central metabolism and cofactor-regeneration pathways, underscoring the potential of systems-level metabolic redesign for industrial-scale NP production. This strategy not only eliminates dependence on the original host organisms but also allows precise control over yield and synthetic routes via gene editing and metabolic engineering (Yang et al., 2025). Nevertheless, the development of microbial cell factories still faces fundamental challenges, primarily due to the limited cellular resources. Introducing heterologous pathways forces the host to balance resources between self-maintenance and foreign product synthesis, often resulting in low production efficiency and strain instability (Zhu and Dai, 2025). Furthermore, genetic and non-genetic heterogeneity within microbial populations can severely impair cell-factory performance at industrial scale (Cao et al., 2024).

Complementing biosynthesis, tandem catalysis in one-pot reactions offers an efficient and innovative approach for structural diversification and modification of natural products. One-pot tandem reactions are defined as “two or more sequential catalytic cycles performed in a single reactor,” (Fogg and dos Santos, 2004) minimizing downstream steps, reducing waste generation, and improving atom economy. For example, rhodium-catalyzed hydroformylation sequences have been employed to directly construct nitrogen-containing heterocycles and alkaloid precursors from olefins, demonstrating the potential of tandem methodologies in fine chemical synthesis (Bondžić, 2015). While this reflects enhanced efficiency in chemical approaches, traditional chemical synthesis still struggles to match the chiral control and step-convergence efficiency of biological systems when constructing complex natural product cores.

From this perspective, the scientific question addressed here is not whether biology or chemistry is superior, but which transformations should be assigned to living cells and which should be delegated to one-pot chemical or chemoenzymatic operations. Microbial biosynthesis is most powerful when multiple stereocenters, protective-group-free redox steps, and highly oxidized or cyclized cores must be assembled from simple carbon sources. In contrast, one-pot tandem chemistry is most valuable when the target requires late-stage halogenation, C-H functionalization, cross-coupling, cycloaddition, dynamic kinetic resolution, or other transformations that are rare, slow, toxic, or poorly evolvable in cells. The conceptual framework of this review therefore defines bio-chemical integration as a division-of-labor strategy: biological modules provide convergent construction of complex “interface molecules,” whereas chemical modules provide divergent access to structurally expanded analog libraries.

Consequently, integrated strategies that merge biological and chemical strengths represent the most cutting-edge research direction. The core idea is to utilize engineered microbial cell factories to efficiently and specifically assemble the chiral core scaffolds of natural products from renewable feedstocks; subsequently, biocompatible chemical or enzymatic reactions are applied to diversify these core intermediates, rapidly generating structurally rich “natural product-like” libraries. This integration is not a simple step-wise connection but requires deep compatibility between the two paradigms at the molecular interface and reaction conditions. Recent breakthrough advances highlight the tremendous potential of this direction. For instance, researchers successfully combined photocatalysts with ene-reductases to develop a cooperative chemo-enzymatic system capable of stereoconvergent reduction of E/Z isomeric mixtures, affording chiral products with high yield and enantioselectivity superior to any single catalyst alone (Litman et al., 2018). This demonstrates that, through ingenious design, artificial chemical catalysts and natural enzymes can cooperate within the same reaction system to accomplish transformations unattainable by either method independently.

Deeper integration even attempts to reconstruct the self-organizing and regenerative properties of living systems in a cell-free environment. For example, Giaveri et al. (2024) coupled a CO₂-fixing metabolic cycle with a cell-free protein synthesis system to create a partially self-synthesizing biochemical network capable of producing glycine from CO₂ and incorporating it into target proteins according to DNA instructions, thereby achieving *in vitro* coupling of metabolism and genetic information systems. Such highly integrated cell-free systems provide an unprecedented platform for studying complex biosynthetic pathways under controlled conditions and seamlessly interfacing with non-natural chemical catalytic steps.

In summary, the combination of synthetic-biology-driven “living factory” biomanufacturing and “one-pot” chemical tandem catalysis represents a paradigm shift in natural pharmaceutical R&D and production. This review explores the synergistic potential of synthetic biology and one-pot chemical tandems in advancing natural-product-based drug discovery and sustainable manufacturing. We systematically elaborate on the construction and optimization principles of engineered strains as living factories, the mechanisms and classifications of one-pot chemical tandem reactions, and critically assess the latest integrated workflows combining fermentation-based biotransformation with chemical catalytic modification, concluding with an outlook on challenges and future trends in this interdisciplinary field. Accordingly, the following sections are organized around three questions: how to build robust microbial factories for natural-product cores; how to select one-pot strategies that are mechanistically compatible with these cores; and how to connect the two through interface-molecule design, spatiotemporal separation or coupling, and cell-free platforms.

## 2 Synthetic biology: constructing efficient “microbial cell factories”

Microbial cell factories represent the engineered realization of synthetic biology principles, aiming to transform living microorganisms into “living factories” capable of efficiently and stably producing target compounds. Building such a factory is far from a simple gene transfer; it is a systematic engineering endeavor following the logic of “chassis selection, pathway construction, system optimization, and performance enhancement.” Each component requires careful design and optimization to truly construct an efficient microbial cell factory.

### 2.1 Selection and optimization of chassis cells

The “chassis cell” is the foundational infrastructure of a cell factory. As the host for biomanufacturing, its choice and performance directly determine the upper limit of the entire factory. Ideal chassis criteria include high growth rate, strong environmental robustness, well-characterized genetic background, and ease of engineering. Traditional model microorganisms such as *E. coli* and *S. cerevisiae*, owing to their mature genetic tools and deep physiological and metabolic understanding, have long been the “workhorses” of cell factories (Liu et al., 2022). However, for many complex natural products originating from actinomycetes or filamentous fungi (e.g., polyketides, non-ribosomal peptides), these model hosts often lack specific precursor supply, cofactors, or post-translational modification systems, failing to meet ideal standards (Liu et al., 2022). Therefore, chassis selection tailored to specific products has become crucial, driving the development and application of non-model microbial chassis. For instance, *Bacillus subtilis*, due to its exceptional protein secretion capacity and generally recognized as safe (GRAS) status, has emerged as a star chassis for industrial enzymes and functional proteins (Zhang Q. et al., 2021). Streptomycetes, being natural producers of numerous antibiotics, possess rich secondary metabolic enzyme systems and regulatory networks, making them ideal platforms for heterologous expression of complex NP gene clusters from other microbes (Yan et al., 2025). Pseudomonads, with their strong tolerance to organic solvents and toxic intermediates, are suitable for producing certain compounds stressful to cells (Liu et al., 2022). Extremophiles such as acidophilic thermophiles also offer possibilities for production under harsh industrial conditions (Liu et al., 2022). The key to chassis selection lies in rationally evaluating the “compatibility” between its metabolic network and the target product synthesis pathway.

After selecting a chassis, deep engineering is required to enhance its performance. Genome reduction is one of the most fundamental strategies, involving deletion of redundant genes unrelated to target production, such as mobile genetic elements or prophage fragments, to reduce metabolic burden, improve genetic stability, and enhance predictability (Sun et al., 2025; Zhang Y. et al., 2024). For example, Zhang Y. et al. (2024) successfully constructed a more stable and safe chassis by deleting two lysogenic phage islands (totaling ~109 kb) from the *Pseudomonas putida* genome. Furthermore, addressing strain instability challenges during industrial scale-up—such as fermentation yield fluctuations up to ±10%, plasmid loss due to metabolic burden during prolonged fermentation, or silent mutations—researchers have begun to enhance strain robustness in long-term fermentation through strategies like eliminating active transposases from the genome or forcibly coupling product synthesis pathways with essential growth genes. Zhu and Dai (2025) precisely reprogrammed global resource allocation in *Bacillus* by constructing synthetic transcriptional switches targeting RNA polymerase, enabling intelligent switching between growth and production modes and significantly improving target product synthesis efficiency.

### 2.2 Heterologous reconstruction and optimization of complex biosynthetic pathways

After chassis selection, the core determinant of production efficiency lies in how to efficiently and precisely control the heterologous biosynthetic pathway. Successful heterologous expression of multi-gene complex NP pathways relies on efficient large-fragment DNA assembly techniques. Modular design of pathways is crucial, wherein the entire pathway is decomposed into functionally independent modules—such as precursor supply, core catalysis, and modification/export modules—facilitating individual optimization, replacement, and combinatorial debugging.^[20]^ For rate-limiting steps within the pathway, protein engineering tools like directed evolution and rational design are powerful means to enhance key enzyme activity, stability, or alter substrate specificity. For instance, engineering domains of polyketide synthases (PKS) or cytochrome P450 oxidases can modify product structural diversity or improve catalytic efficiency (Chen et al., 2016; Zhang J. et al., 2024). Recent work on P450 enzymes and their reductase interface via directed evolution boosted catalytic activity for serotonin synthesis by over 36-fold, significantly increasing final product yield (Xu et al., 2025).

However, this is merely the first step in creating efficient cellular factories. Successfully implanting highly optimized static modules into chassis cells does not guarantee they will automatically collaborate optimally within complex cellular environments. Traditional “strong promoter continuous drive” expression strategies often trigger persistent “resource competition” between heterologous pathways and the host’s intrinsic growth metabolism. This leads to metabolic imbalance, accumulation of intermediate products, ultimately limiting yield and compromising strain stability (Boyle and Silver, 2012; He et al., 2016; Renda et al., 2014). Consequently, modern synthetic biology has shifted its focus from static “overexpression” to dynamic “precision regulation.” Its core objective is to transform exogenous pathways from isolated machines operating at full throttle—incongruous with cellular systems—into integrated smart factories capable of sensing cellular states, adapting dynamically, and intelligently coordinating with the host. This marks our transition from engineering pathway components to a new phase of systematic pathway manipulation across temporal and spatial dimensions.

The core of dynamic regulation lies in enabling pathway expression to respond to specific signals within and outside the cell. Metabolite-responsive regulation utilizes natural or artificially engineered transcription factor biosensors to convert concentration changes of key metabolites (pathway precursors, intermediates, or end products) into switching or intensity-modulating effects on gene expression. For instance, researchers engineered ligand-binding domains to develop biosensors highly sensitive to coumaric acid, a natural plant metabolite precursor, enabling self-feedback regulation of downstream synthetic pathways (de Frias et al., 2018). Another mainstream strategy is growth-stage coupled regulation. By designing genetic circuits based on quorum sensing, nutrient depletion, or other growth-stage markers, these systems suppress product synthesis during the exponential growth phase to ensure biomass accumulation. Upon entering the stationary phase, the production modules automatically activate. This effectively decouples resource competition between growth and production and has been widely applied in constructing high-yield strains for various antibiotics and secondary metabolites (Yook et al., 2025).

**Figure 1:**
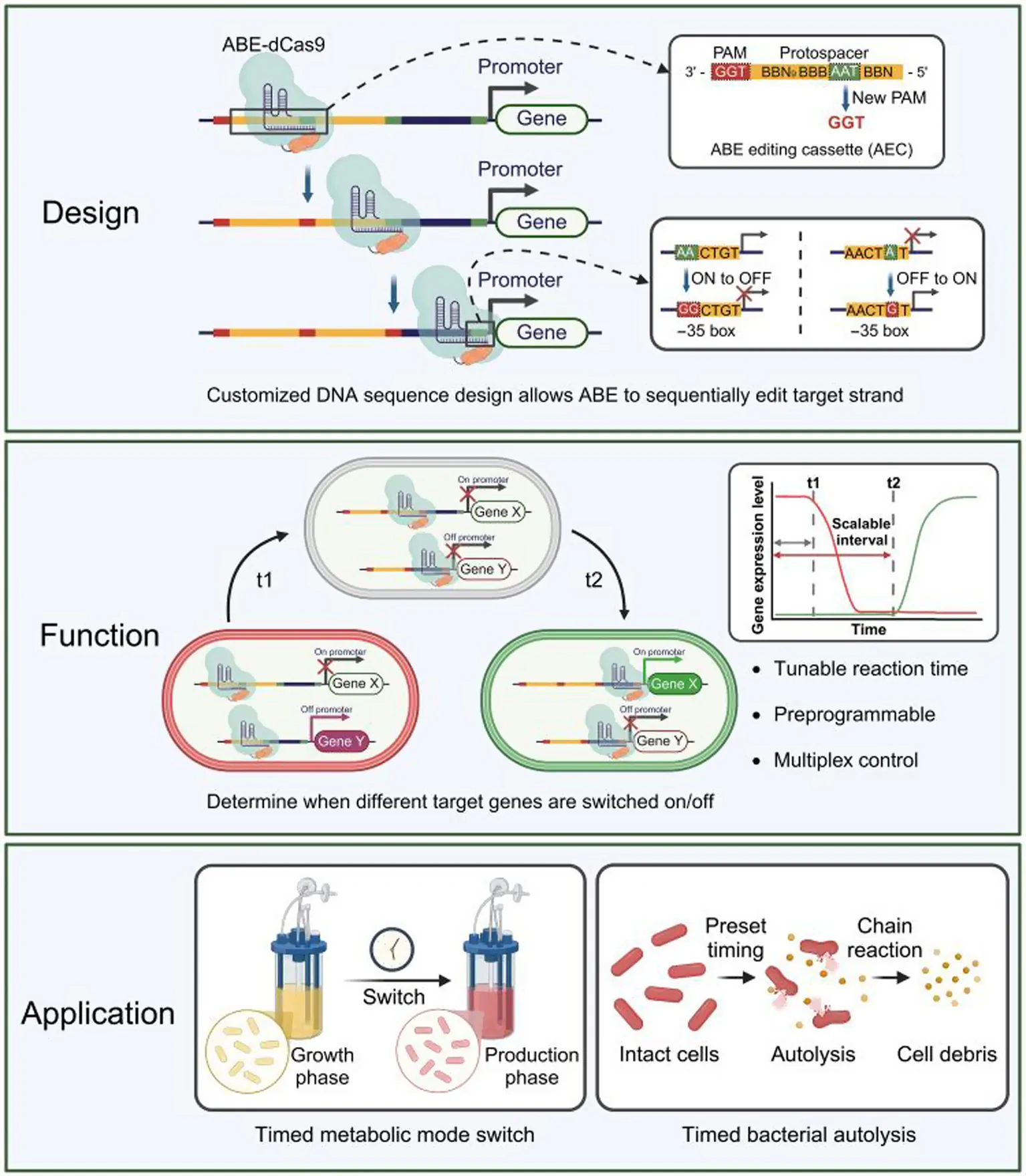
Schematic of BioFuse: a programmable timer switch of gene expression. The design panel shows how adenine base editors and programmable tandem boxes create timed promoter switching; the function panel illustrates sequential ON/OFF gene expression; and the application panel shows timed metabolic-mode switching and bacterial autolysis. Source: Reproduced from Huang C. et al. (2025), licensed under CC BY 4.0.

The most cutting-edge regulatory paradigms have now entered the realm of spatiotemporal precision control. In temporal control, emerging optogenetic switches enable researchers to precisely time gene expression from minutes to hours using non-invasive light signals, providing unprecedented manipulation tools for studying metabolic kinetics (Sagharyan et al., 2025). As shown in Figure 1, Tian et al. developed the “BioFuse” system, innovatively utilizing single-base editors and programmable DNA tandem boxes to achieve precise gene temporal regulation over hours to days without exogenous inducers. This system successfully decoupled the lycopene synthesis pathway from *E. coli*’s growth phase. To silence the pathway during growth-dominant periods to reduce metabolic burden, then automatically sequentializing synthetic modules once biomass became abundant, significantly boosting final yield (Huang C. et al., 2025). This achievement demonstrates the immense potential of endogenous programming for optimizing metabolic timing. In spatial organization, researchers constructed synthetic protein scaffolds or artificial organelles to co-localize multiple enzymes from a pathway in specific sequences and proportions within designated cellular regions, such as the cell membrane or engineered bacterial microcompartments (Sun et al., 2025). This “metabolic compartmentalization” not only significantly enhances metabolic flux by shortening the diffusion distance of intermediates, an effect known as “substrate channeling,” but also effectively isolates metabolic intermediates that are toxic to the host cell. Tools for achieving such precise spatial assembly include synthetic and orthogonal protein interfaces, such as short peptide–based interaction pairs (e.g., SpyTag/SpyCatcher), synthetic coiled coils, and split inteins (Kim et al., 2025). These interfaces function as standardized “biological connectors,” overcoming compatibility limitations of natural enzyme complexes to assemble catalytic modules from diverse sources into functional “nanomachines” on demand (Kim et al., 2025).

**Figure 2:**
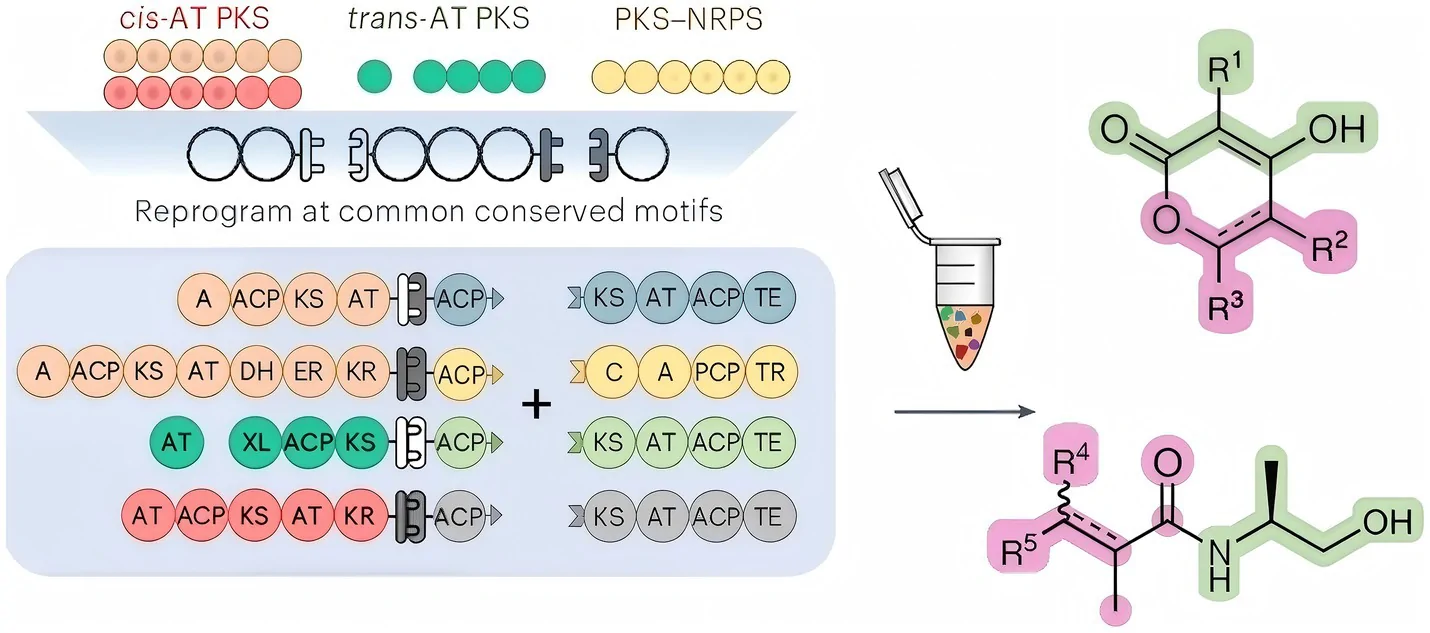
Plug-and-play reprogramming of modular PKSs the figure summarizes how conserved docking or communication motifs can be redesigned to connect cis-AT, trans-AT, and PKS-NRPS modules, enabling combinatorial assembly and diversification of polyketide carbon skeletons. Source: Reproduced from Huang Z. et al. (2025) with permission with Springer Nature.

**Figure 3:**
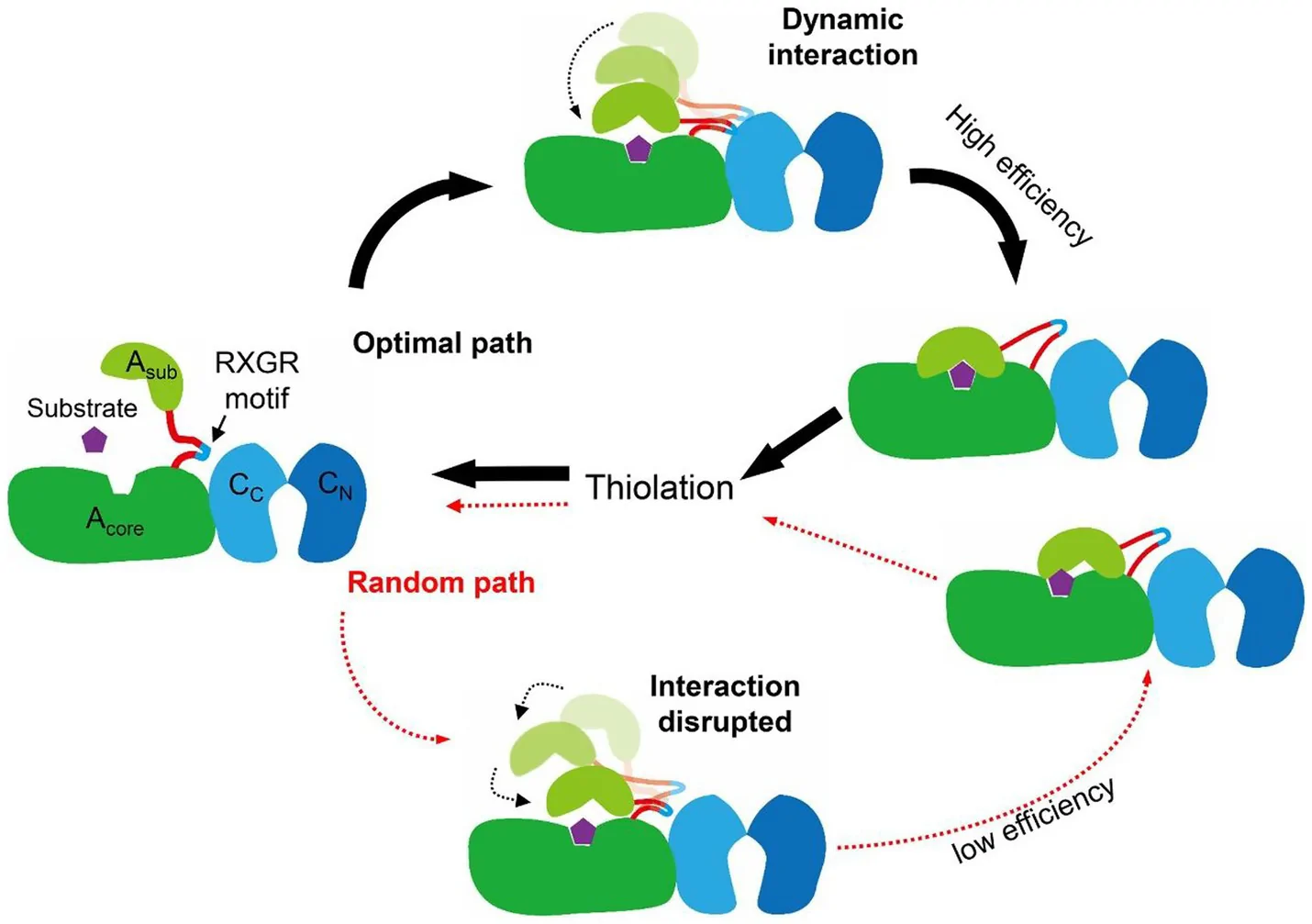
A model for the control mechanism of the adenylation activity in an NRPS module, mediated by dynamic interaction between the A and C domains. The diagram highlights the productive dynamic interaction between the adenylation and condensation domains, the optimal thiolation path, and the loss of efficiency when the interface is disrupted, thereby explaining why domain communication is central to NRPS reprogramming. Source: Reprinted from Peng et al. (2024) with permission from Elsevier.

The concept of spatially reorganizing enzymes is particularly crucial in the engineering of giant modular synthases, such as polyketide synthases (PKS) and nonribosomal peptide synthetases (NRPS). These enzymes inherently possess assembly line-like modular architectures; however, the specific interactions between their native modules, mediated by docking domains, impose significant constraints on module interchangeability and recombination (Peng et al., 2024). As shown in Figure 2, Zhang et al. engineered and replaced these interaction interfaces to create more broadly compatible, plug-and-play enzyme modules, thereby enabling the customized biosynthesis of complex natural product carbon skeletons (Huang Z. et al., 2025). Furthermore, as shown in Figure 3, the study by Peng et al. (2024) provided an in-depth analysis of the dynamic interaction interface between the adenylation domain and the condensation domain within NRPS modules. This work offers key insights for the rational reprogramming of these “molecular assembly lines” to produce novel peptide structures.

In summary, through modular design, protein engineering, and ultimately with dynamic and spatiotemporally precise regulatory strategies, we can construct highly efficient cellular factories in the laboratory. However, the ultimate goal of research is industrialization. In industrial production, ensuring that the sophisticated designs developed in the laboratory maintain their performance and robustness during dynamic scale-up processes represents a major challenge.

### 2.3 Scale-up challenges and systems biology solutions

Translating strains constructed in shake flasks into stable and cost-effective industrial production lines is a bioreactor-engineering and evolutionary-stability problem, not only a genetic-design problem. Shake flasks and microwell cultures usually provide short diffusion distances and relatively homogeneous exposure to oxygen and nutrients, whereas pilot and production fermenters generate spatial gradients in dissolved oxygen, carbon source, pH, carbon dioxide, temperature, and shear. For aerobic cell factories, oxygen transfer is often limiting because oxygen has low solubility in aqueous broth and the oxygen transfer rate depends on the volumetric mass-transfer coefficient, gas–liquid interfacial area, agitation, sparging, broth viscosity, and cell oxygen-uptake rate (García-Ochoa and Gómez, 2009). In large tanks, circulation times may be long enough that cells repeatedly move through oxygen-rich, oxygen-poor, substrate-rich, and substrate-depleted zones. These oscillations change NADH/NAD + balance, ATP availability, overflow metabolism, stress-response transcription, and product export; therefore, a strain that is optimal at 250 mL scale can lose productivity even if biomass formation remains high (Crater and Lievense, 2018).

A quantitative scale-down case illustrates the magnitude of the problem. In a recombinant *Corynebacterium glutamicum* fed-batch process for cadaverine, a two-compartment scale-down reactor that imposed pH, glucose, and oxygen gradients reduced cadaverine production by approximately 26, 49, and 59% as the average plug-flow residence time increased from 1 to 2 and 5 min, respectively, compared with the homogeneous control, while biomass production was not proportionally reduced (Olughu et al., 2020). This example shows that scale-up losses may arise from altered physiology, viable-but-non-culturable subpopulations, and by-product formation rather than simple cell death. Similar considerations apply to high-value natural-product cell factories, where oxygen-dependent P450 tailoring steps, acetyl-CoA or malonyl-CoA supply, NADPH regeneration, and membrane transport are frequently sensitive to oxygen transfer and carbon-feed heterogeneity.

Future development of cellular factories will increasingly rely on interdisciplinary system integration. This involves the deep integration of computational predictions from genome-scale metabolic models (GEMs), high-throughput automated library construction and screening platforms, and artificial intelligence/machine learning (AI/ML) algorithms to form an intelligent “Design-Build-Test-Learn” (DBTL) closed loop (Sun et al., 2025). AI can be used to predict optimal gene editing targets, design high-performance enzyme components, and even mine promising novel biosynthetic pathways from vast numbers of cryptic gene clusters. This has the potential to significantly shorten the multi-year timeline of strain development and enable the discovery and reconstruction of biosynthetic capabilities for complex natural products (Liu et al., 2022). Consequently, robust scale-up requires scale-down screening, not only end-point titer optimization. Modern scale-down systems, including two-compartment reactors, plug-flow recirculation loops, and microfluidic single-cell simulators, deliberately reproduce dissolved-oxygen, pH, and feed gradients to identify failure modes before pilot fermentation. These platforms can be coupled with online off-gas analytics, Raman or mass-spectrometry metabolite monitoring, CFD-based mixing models, and multi-omics readouts to define process design spaces rather than single flask-derived optima (Arulrajah et al., 2025; Du et al., 2022).

However, the biomanufacturing capabilities of synthetic biology still face limitations when pursuing structural diversification. On the one hand, the high specificity of enzyme catalysis ensures synthetic precision but also constrains the ability to modify non-natural sites or introduce entirely new chemical functional groups. On the other hand, the metabolic burden and physiological limits of cells make it extremely difficult to operate multiple competing pathways in parallel within a single organism to produce libraries of structural analogs. Consequently, when the research objective shifts from efficiently producing a single molecule to rapidly creating diverse libraries of lead compounds, purely biosynthetic strategies often fall short of achieving the desired outcome (Table 1).

**Table 1:** Major natural-product classes and where bio-chemical integration provides the greatest advantage.

Natural-product class	Typical biosynthetic bottleneck	Chemical or chemoenzymatic opportunity	Representative integration advantage
Terpenoids	IPP/DMAPP flux, P450 oxygenation, hydrophobicity or volatility, and product toxicity	Late-stage oxidation, epoxidation, cyclopropanation, photoredox or artificial metalloenzyme transformations	Cells build hydrocarbon or oxygenated cores; chemistry expands oxidation and carbocycle motifs beyond native terpene tailoring (Yang et al., 2025; Huang et al., 2021)
Alkaloids	Strict stereochemical control, toxic amines or iminium intermediates, and N- or O-methylation balance	Pictet-Spengler reactions, Ullmann coupling, C-H amination, and dynamic kinetic resolution	Enzymes set chiral amine centers; chemical steps close or arylate heterocycles to access analogs (Zhang F. et al., 2021; Lei et al., 2024)
Polyketides	Very large PKS genes, extender-unit supply, module incompatibility, and uncertain docking-domain communication	C-H functionalization, glycosylation, halogenation, cross-coupling, and module-interface redesign	Biosynthesis assembles carbon skeletons; chemistry or engineered interfaces diversify positions poorly reached by PKS editing (Ray et al., 2024; Zhang J. et al., 2024; Chen et al., 2016; Kim et al., 2025; Huang Z. et al., 2025; Yao S. et al., 2025)
Non-ribosomal peptides	NRPS domain communication, epimerization, macrocyclization, and incorporation of rare amino acids	Late-stage macrocyclization, lipidation, heterocycle formation, and noncanonical amino acid incorporation	Biological assembly preserves sequence logic; chemical handles broaden residue diversity and ring architectures (Kim et al., 2025; Peng et al., 2024; Liu et al., 2023)
Flavonoids and phenolics	Precursor competition, regioselective hydroxylation, glycosyl-donor supply, and poor solubility	One-pot enzymatic glycosylation, oxidative coupling, acylation, and multicomponent derivatization	Fermentation supplies phenylpropanoid cores; tandem enzymes generate glycoside libraries quickly (de Frias et al., 2018; Yao et al., 2024)

## 3 One-pot tandem chemistry: efficient synthesis and diversification methods

In this condition, chemical synthesis, particularly one-pot tandem catalysis, demonstrates the ability to precisely modify and derivatize the core scaffolds obtained from biosynthesis, owing to its generality across reaction types and high flexibility in functional group transformations.

### 3.1 Core principles and advantages of one-pot tandem catalysis

The essence of one-pot tandem catalysis extends beyond operational simplification; it is an information-management strategy in which the molecular information generated in one step is immediately consumed by the next step before the intermediate decomposes, racemizes, or requires isolation (Bai and Jiang, 2023; Nicolaou and Chen, 2009). For readers from microbiology, the analogy is a metabolic pathway compressed into one reactor: intermediates are transmitted between catalytic modules, but the modules may be small-molecule catalysts, metals, organocatalysts, enzymes, or light-activated species rather than enzymes encoded by genes. Based on how and when the catalytic modules are activated, one-pot syntheses can be categorized into three operational modes: domino reactions, multicomponent reactions, and sequential one-pot syntheses (Fogg and dos Santos, 2004; Ma and Zhang, 2022; Table 2).

**Table 2:** Mechanistic comparison of one-pot strategies relevant to natural-product diversification.

Strategy	Mechanistic logic	Typical catalysts and conditions	Natural-product modification relevance
Domino/tandem	A functional group generated in one step spontaneously triggers the next transformation without isolation.	Organocatalysts, transition metals, photoredox catalysts, acids or bases; usually one solvent and one temperature.	Rapid formation of rings, cascaded redox-cyclization, and skeletal rearrangement.
Multicomponent	Three or more starting materials converge into a single product through coupled bond-forming events.	Brønsted or Lewis acids, organocatalysts, and metal catalysts; moderate temperatures and polar solvents are often tolerated.	Fast analog generation by changing amine, aldehyde, isocyanide, or alkyne components.
Sequential one-pot	Reagents, catalysts, pH, solvent, light, or temperature are changed stepwise in the same vessel.	Enzymes plus metals, lipases plus Ru/Rh catalysts, photocatalysts, or buffer/organic biphasic media.	Useful when biological and chemical catalysts are partially incompatible; avoids intermediate purification.

This classification is important for bio-chemical integration because it determines how much incompatibility can be tolerated. Domino and multicomponent reactions require broad mutual compatibility from the beginning of the reaction, whereas sequential one-pot operations allow the researcher to first run an enzyme-compatible aqueous step and then adjust pH, solvent, temperature, light, or catalyst loading for a chemical finishing step. Thus, the one-pot concept is not a single method but a spectrum of reactor designs linking metabolic-like convergence with chemical diversification.

In the field of natural product synthesis and modification, one-pot tandem catalysis has shown remarkable efficacy due to its ability to efficiently construct complex ring systems and multiple stereogenic centers, leading to numerous landmark applications (Nicolaou and Chen, 2009). For instance, in the total synthesis of the anti-influenza drug Oseltamivir, researchers efficiently constructed its core ring system and multiple chiral centers through three one-pot operations. Compared with traditional linear syntheses, this approach significantly shortened the synthetic route, demonstrating substantial efficiency advantages in the manufacturing of complex drug molecules (Ishikawa et al., 2009). Tandem strategies also play a crucial role in constructing core carbon skeletons of natural products. A recent illustration is the strategic comparison of daphenylline syntheses, in which Diels-Alder, Michael addition, oxidative, radical, and C-H functionalization cascades were used to access congested Daphniphyllum alkaloid frameworks, highlighting how modern tandem logic accelerates polycyclic skeleton construction (Zhao and He, 2025).

Notably, the concept of one-pot synthesis is not confined to traditional chemical catalysis; it also demonstrates powerful integrative capabilities in the field of biocatalysis. Ye et al. constructed a multicatalytic one-pot tandem system by combining various enzymes such as glycosyltransferases and polyphenol oxidases. This system enabled the milestone achievement of synthesizing up to 26 phenylethanoid glycoside natural products from a common precursor in a single operation (Yao et al., 2024). This work perfectly illustrates the exceptional potential of the one-pot approach for rapidly generating libraries of natural product analogs. Furthermore, by integrating diverse transformations such as C–H functionalization, cyclization, and coupling, one-pot methodologies also provide modular and diverse synthetic routes for heterocyclic structures commonly found in natural products, such as quinolines and tricyclic compounds, making them highly suitable for late-stage, in-depth modifications of core scaffolds (Ma and Zhang, 2022).

In summary, one-pot tandem catalysis enables efficient and flexible modifications of the core scaffolds obtained from biosynthesis. It serves as a key chemical tool for rapidly generating structurally diverse libraries of natural product-like compounds from a single biological precursor (Nicolaou and Chen, 2009). This also lays a theoretical foundation for overcoming the compatibility challenges between chemical and biological systems and achieving truly synergistic tandems. However, to translate one-pot tandem catalytic reactions into methodologies for the precise modification of natural products, it is essential to investigate which types of tandem reactions are applicable. Consequently, scientists have conducted extensive research in this area.

### 3.2 Tandem reaction types suitable for natural product modification

For microbiology-oriented readers, the key mechanistic feature of these transformations is that each chemical step substitutes for an enzymatic function that is difficult to encode or regulate in a cell. Redox sequences mimic tailoring oxidases and reductases but can access non-natural oxidation states; C-H functionalization mimics late-stage hydroxylation or amination without requiring a matching P450; and DKR mimics biological stereochemical proofreading by continuously racemizing the undesired enantiomer while an enzyme selectively consumes the preferred one.

First, the redox sequence is a key strategy for precisely remodeling carbon skeletons and introducing functional groups. Its design logic lies in combining complementary oxidation and reduction steps to achieve sequential modifications at specific molecular sites (Zhang F. et al., 2021). A classic example is the biomimetic synthesis of the antimalarial drug artemisinin. In this process, researchers simulated the biological oxidation pathway within the plant by subjecting the artemisinic acid precursor to light and a photosensitizer. Through a multi-step oxidative tandem reaction involving singlet oxygen mediated cycloaddition and subsequent intramolecular rearrangement, the characteristic peroxy bridge, a crucial pharmacophore of artemisinin, was constructed in a single step (Yu and Wen, 2011). This route avoids the use of multi-step, harsh traditional chemical oxidants, demonstrating the exceptional efficiency of tandem reactions in biomimetic synthesis. In the synthesis of complex indole alkaloids, the iminium ion intermediate generated from amine oxidation can undergo an immediate intramolecular Pictet-Spengler cyclization, thereby constructing core heterocyclic skeletons such as tetrahydro-*β*-carbolines in a one-pot manner, providing a concise route for rapidly assembling natural product libraries.

**Figure 4:**
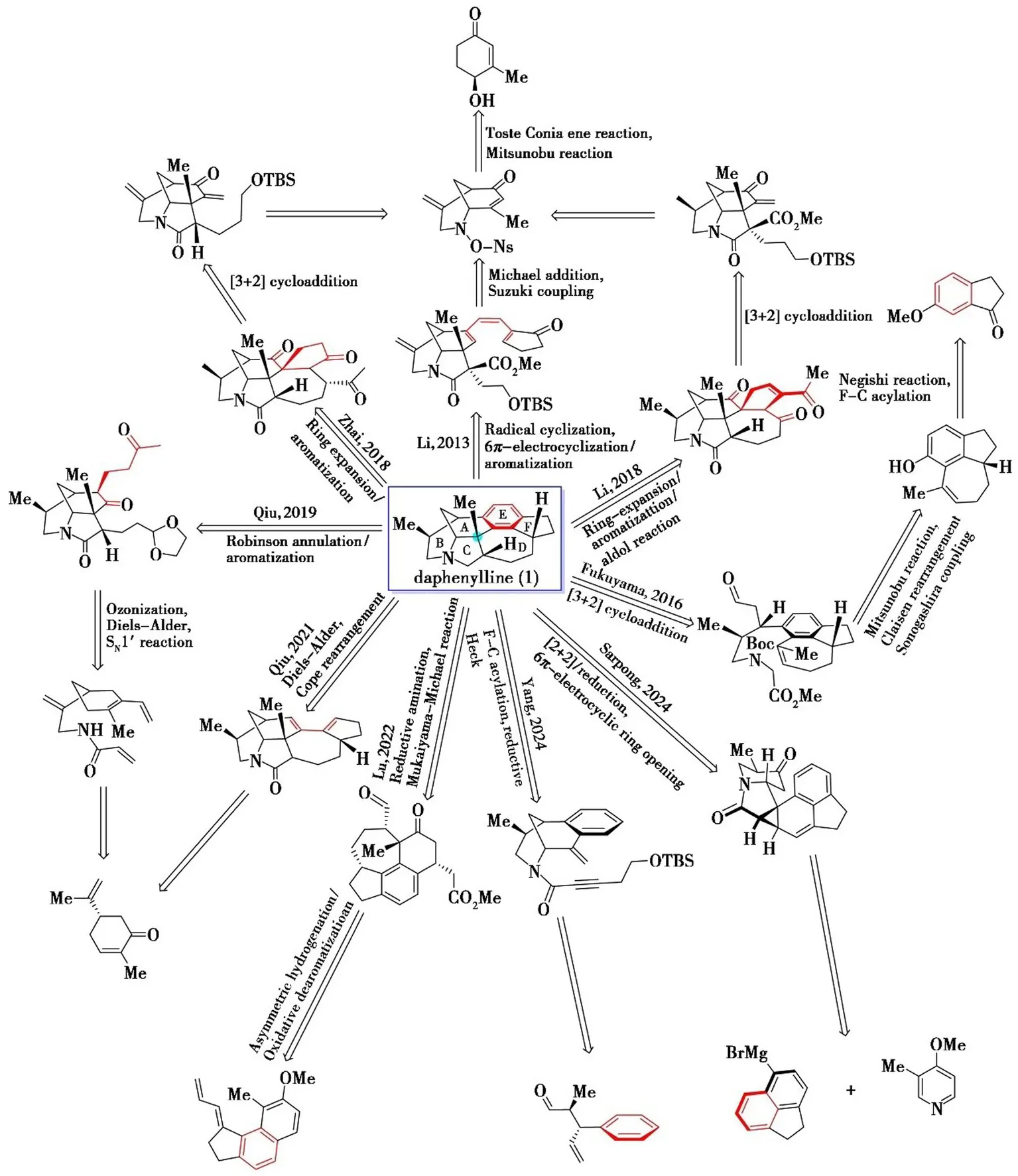
Summary of synthetic strategies for Daphenylline. The scheme compares representative cascade and C-H functionalization strategies used to assemble the congested Daphniphyllum alkaloid framework, highlighting how tandem bond-forming events reduce step count and support rapid access to polycyclic natural-product scaffolds. Source: Reproduced from Zhao and He (2025), licensed under CC BY 4.0.

Second, cyclization tandems initiated by direct C-H bond functionalization represent the pinnacle of atom economy in modern synthetic chemistry. This strategy bypasses the cumbersome traditional steps of pre-functionalization by directly utilizing inert C-H bonds as reactive sites. Through transition metal catalysis (e.g., palladium, rhodium), these bonds are activated to trigger subsequent cyclization or coupling reactions. For example, via an ortho C–H amination and cyclization tandem, the tetrahydroisoquinoline skeleton, a core structure found in numerous bioactive alkaloids such as erythromycin, can be directly and efficiently constructed from simple phenethylamine derivatives (Zhang F. et al., 2021). More groundbreakingly, as shown in Figure 4, Liu et al., through substrate design, achieved a continuous intramolecular C-H functionalization process capable of forming multiple carbon–carbon bonds in one step, rapidly assembling complex polycyclic skeletons of natural products, such as the key cyclization step in the synthesis of the Daphniphyllum alkaloid daphenylline (Zhao and He, 2025). This direct transformation from C-H bonds to complex ring systems significantly enhances the efficiency of synthesizing complex natural products and their analogs.

Finally, dynamic kinetic resolution (DKR) is an ultimate solution to the challenge of chiral molecule synthesis, perfectly aligning with the theme of bio-chemical synergy. DKR is not a single reaction type but rather an ingenious strategy that synergistically employs both metal catalysts and biocatalysts. Metal catalysts such as ruthenium or rhodium complexes rapidly racemize alcohol or amine substrates, while highly selective enzymes such as lipases or ketoreductases specifically convert one enantiomer. This enables, in theory, the production of optically pure compounds in 100% yield (Chen et al., 2019; Zhou et al., 2026). This strategy integrates the high efficiency of chemical catalysis with the high stereospecificity of biocatalysis and has been successfully applied in the kilogram-scale preparation of various chiral drug intermediates, such as those for certain beta-blockers and antiviral drugs (Yu B. et al., 2025). The successful implementation of DKR not only addresses the challenge of chiral control in purely chemical synthesis but also expands the application of biocatalysis in non-aqueous or non-physiological conditions. It stands as a paradigm of molecular-level synergy between chemical methods and biological tools.

In summary, the three types of one-pot strategies—redox sequences, C-H functionalization/cyclization tandems, and dynamic kinetic resolution—provide a powerful foundation for the late-stage modification and derivatization of natural products from three distinct dimensions: precise functional group transformation, efficient skeleton construction, and stereogenic center control. Their common feature is the condensation of multiple reaction steps, reducing losses and waste generated from isolation and purification processes. This establishes a robust chemical methodology foundation for rapidly converting universal biosynthetic precursors into diverse libraries of “natural product-like” compounds.

### 3.3 Limitations of chemical synthesis and compatibility with biological systems

Although one-pot tandem chemistry demonstrates remarkable efficiency in constructing complex molecules *in vitro*, it still presents several limitations. The primary and most pervasive challenge lies in the severe incompatibility of reaction conditions. Efficient chemical catalysis, particularly processes involving transition metals (such as palladium, rhodium, and ruthenium) for C–H activation or cross-coupling reactions, often requires high temperatures (above 70 °C), anhydrous environments, organic cosolvents (e.g., toluene, THF), or extreme pH levels (Chen et al., 2025). However, these conditions are often detrimental to maintaining enzyme activity and cell viability. For example, the classic ruthenium catalysts employed in dynamic kinetic resolution (DKR) require temperatures above 70 °C to activate their efficient hydrogen-transfer-based racemization mechanism (Park et al., 2025; Verho and Bäckvall, 2015). This inherently restricts the range of compatible enzyme partners to a few extremely thermostable lipases (such as CALB), excluding a vast number of proteases or oxidoreductases that exhibit excellent selectivity at ambient temperatures but poor thermal stability. Similarly, the reaction media for many metal-catalyzed transformations are incompatible with the aqueous environment of biological systems, as strongly polar organic solvents can cause protein denaturation or cell membrane disruption. Therefore, developing biocompatible catalysts that retain high activity under physiological temperatures, neutral pH, and in aqueous or biphasic media is key to addressing this issue. This incompatibility should be treated as a multi-parameter design problem rather than as a simple yes-or-no assessment.

Second, mutual catalyst inhibition constitutes a core stability challenge in integrated systems. This conflict is particularly pronounced in chemoenzymatic DKR. On one hand, chemical catalysts such as metal ions, organic ligands, or nanoparticles can irreversibly occupy the active sites of enzymes or disrupt their critical disulfide bonds through redox reactions, leading to enzyme deactivation (Liao et al., 2019; Wang et al., 2024). On the other hand, the complex and diverse components present in biological systems can also deactivate chemical catalysts. For instance, thiols, phospholipids, or nucleic acid fragments released during cell lysis can bind strongly to the surfaces of metal catalysts, rendering them catalytically inactive (Liao et al., 2019; Wang et al., 2024). Certain stabilizers or surfactants added to enzyme preparations may also inhibit the reactivity of metal centers. A successful DKR system must ensure that the racemization catalyst and the enzyme coexist peacefully and maintain their functions over extended periods, which requires sophisticated engineering of their interface (Wang et al., 2024).

Finally, the biocompatibility of substrates and products restricts the selection of “interface molecules.” Reagents used in chemical steps, such as strong oxidants or organometallic compounds, or the intermediates and products generated, may be toxic to living cells, interfering with normal metabolic pathways, membrane potential, or gene expression. Even in relatively mild post-fermentation modifications, if the target chemically modified product itself possesses antimicrobial or cytotoxic properties, its reintroduction into living cells for the next round of biotransformation can be hindered. This necessitates a prospective evaluation of the potential impact of every chemical reagent and product involved in each step on any subsequent biological components that may be utilized when planning an entire “bio-chemical” tandem pathway.

## 4 Synergistic tandems of biosynthesis and chemical catalysis

### 4.1 Logic and design principles of synergistic strategies

The rise of the “bio-chemical synergy” strategy stems from a profound understanding of the intrinsic characteristics of the two catalytic systems and the complementary advantages between them. Its core logic lies in utilizing the “living factories” constructed by synthetic biology to efficiently and stereospecifically synthesize structurally complex core scaffolds or versatile intermediates of natural products from inexpensive, renewable feedstocks. These intermediates are then subjected to flexible and diversified late-stage functional group modifications and skeletal remodeling via synthetic chemistry, particularly biocompatible one-pot tandem catalysis, thereby enabling the rapid construction of structurally diverse libraries of “natural product-like” compounds (Zhang P. et al., 2025; Zhang and Szostak, 2022).

We define three practical integration paradigms. The first is sequential post-fermentation modification, in which cells produce an interface molecule and chemical or enzymatic steps are optimized separately. The second is concurrent one-pot chemoenzymatic catalysis, in which compatible catalysts share a controlled reactor. The third is cell-free bio-chemical integration, in which enzymes, cofactors, chemical catalysts, and DNA templates are combined in an open system. These paradigms differ in complexity, but all rely on matching the chemical handle of the interface molecule to the mode of catalytic diversification.

**Figure 5:**
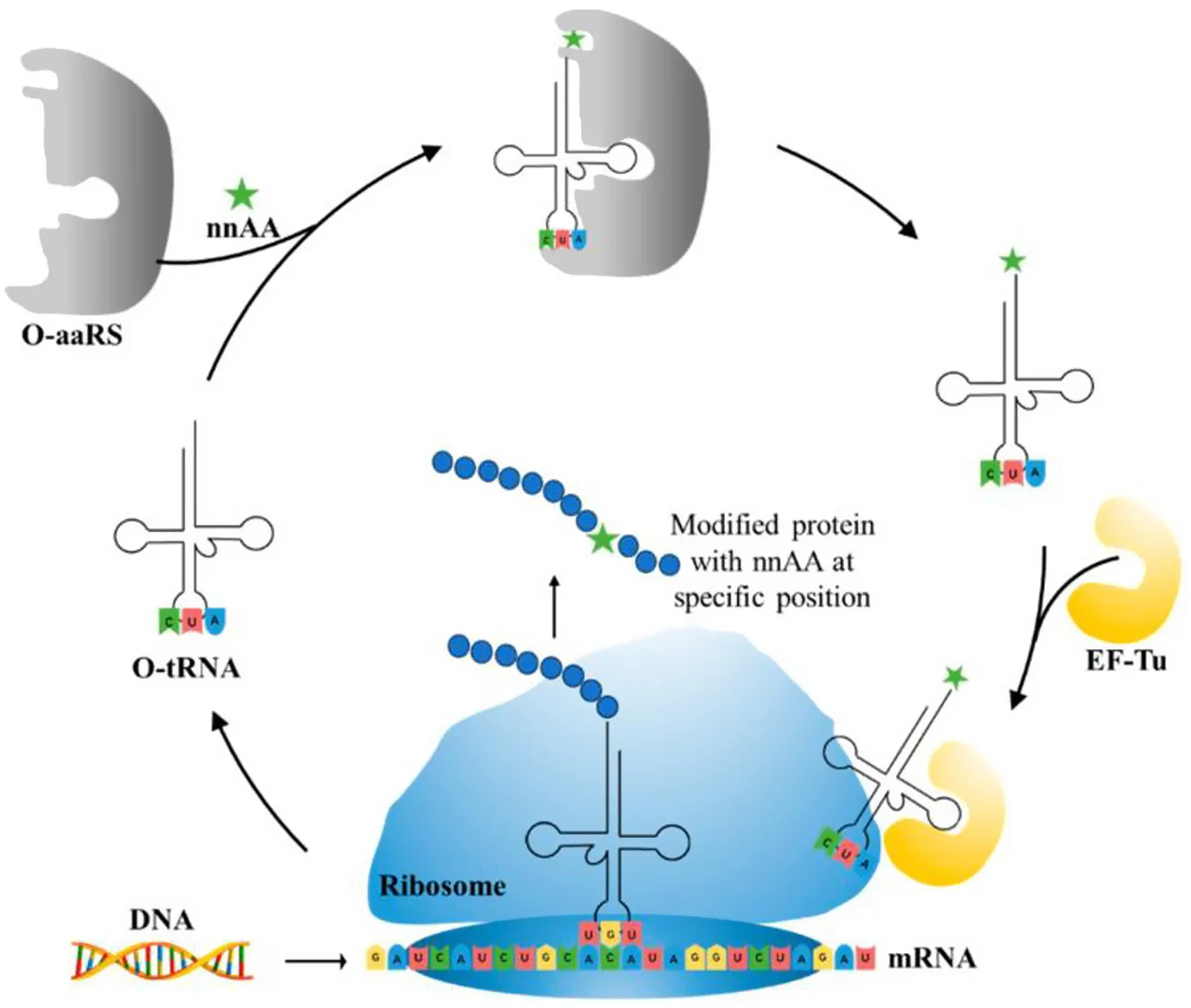
Scheme of site-specific incorporation of non-natural amino acids (nnAAs, represented by stars). O-aaRS represents orthogonal aminoacyl tRNA synthetase; nnAA represents non-natural amino acid; O-tRNA represents orthogonal tRNA; EF-Tu represents elongation factor Tu. Source: Reproduced from Liu et al. (2023), licensed under CC BY 4.0.

The primary design principle for successfully implementing this strategy is the design of the “interface molecule.” An ideal interface molecule must possess dual attributes. From a biological perspective, it must be synthesizable by the host metabolic network with high yield and titer while exhibiting low cytotoxicity. Chemically, it should contain selectively activatable “chemical handles,” such as alkenes, alkynes, specific C-H bonds, hydroxyl groups, or amino groups, to facilitate efficient subsequent derivatization (Hsu et al., 2015; Zhang Q. et al., 2025). In recent years, the design of “unnatural” interface molecules that go beyond the boundaries of natural metabolism has become a cutting-edge focus. As shown in Figure 5, Liu et al. (2023) engineered enzymes or utilized artificial metalloenzymes to incorporate unnatural amino acids or designed cofactors into biosynthetic pathways, creating molecular motifs with unique reactivity that do not exist in nature, thereby opening new avenues for subsequent chemical transformations.

Artificial metalloenzymes provide a direct example of this interface concept. An engineered *E. coli* system combining heterologously expressed terpene-pathway enzymes with an iridium-porphyrin artificial metalloenzyme generated an unnatural product from a biologically produced alkene-containing terpene intermediate, thereby demonstrating that a living cell can host both natural biosynthesis and a new-to-nature metal-catalyzed reaction when cofactor transport, protein scaffold engineering, and cultivation conditions are coordinated (Huang et al., 2021).

A deeper design principle lies in the intelligent regulation of what is termed spatiotemporal coupling. Simple linear tandems, where fermentation is conducted first and then followed by chemical modification, are no longer sufficient to meet the demands of efficient and integrated manufacturing. Cutting-edge research is dedicated to achieving profound integration of the two catalytic modes across both temporal and spatial dimensions (Authors, 2025).

On the temporal dimension, this involves constructing genetic circuits that dynamically couple the expression of key enzymes or tolerance elements required for chemical modification with the biosynthetic phase. For example, when a cellular factory accumulates a specific concentration of an intermediate, it can autonomously induce the expression of membrane transporters to facilitate its export, or express organic solvent-tolerant proteins to prepare for subsequent chemical steps (Ford and Silver, 2015).

**Figure 6:**
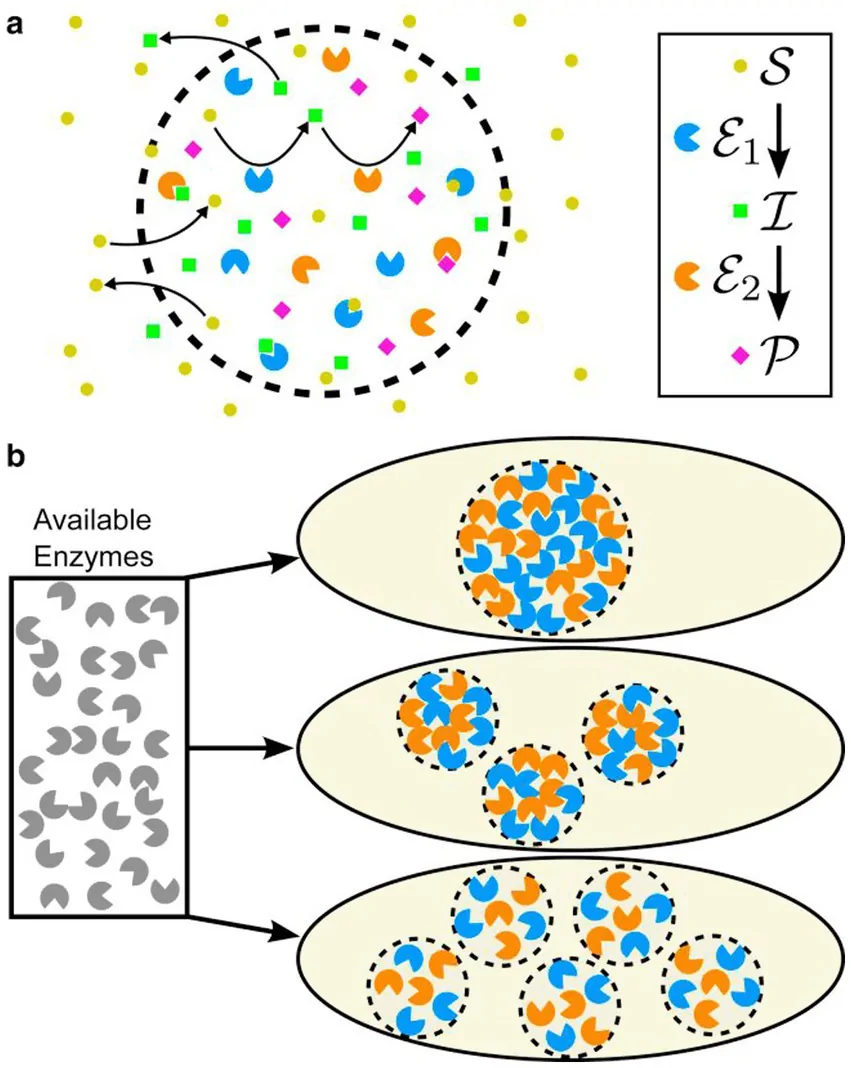
Illustration of the pathway model and compartmentalization strategies. **(a)** Model two-step enzymatic pathway with enzymes contained within a microcompartment. The permeable compartment shell allows for the exchange of metabolites *𝒮* and *ℐ* with the cytoplasm. **(b)** A fixed number of enzymes could be distributed according to many different compartmentalization strategies, each characterized by a particular compartment size, enzyme density, and ratio of *ℰ1* to *ℰ2* enzymes. Each such strategy would lead to a different pathway flux, and therefore a different productivity. Source: Reproduced from Hinzpeter et al. (2017), licensed under CC BY-NC-ND 4.0.

On the spatial dimension, as shown in Figure 6, researchers draw inspiration from the concept of “metabolic compartmentalization” in synthetic biology. By utilizing artificial protein scaffolds, bacterial microcompartments, or liposomes, specific chemical catalysts and enzymes can be co-localized to create “nanoscale integrated reactors.” This spatial proximity not only accelerates intermediate transfer and enhances overall reaction rates but also effectively isolates toxic chemical catalysts from the fragile intracellular environment (Hinzpeter et al., 2017).

Furthermore, systematic computational tools and machine learning have become indispensable components of the design process. From genome-scale metabolic models that predict optimal biosynthetic precursors, to quantum chemical calculations that simulate the feasibility and selectivity of chemical modifications, and to the use of machine learning algorithms to recommend potential bio-chemical reaction combinations from vast literature data, computation-driven rational design has significantly accelerated the construction and optimization of synergistic pathways (Kretschmer and Kortemme, 2022).

In summary, bio-chemical synergy has evolved from a conceptual idea into a comprehensive engineering framework encompassing molecular design, spatiotemporal regulation, and computational assistance. Its goal is to seamlessly integrate the synthetic convergence of biological systems with the transformative divergence of chemical systems, ultimately enabling the precise, efficient, and sustainable manufacturing of diverse functional molecules directly from genetic sequences.

### 4.2 Chemically enabled post-fermentation modification

Chemically enabled post-fermentation modification represents the most mature and widely adopted paradigm within the bio-chemical synergy strategy. This classic approach adheres to a “linear tandem” logic, whose core advantage lies in decoupling the biosynthetic and chemical transformation steps in both time and space, allowing each to operate under its own optimal conditions (El-malek and Steinbüchel, 2022). Microbial fermentation focuses on efficiently and stereoselectively constructing the core scaffold or key precursors of a natural product from renewable feedstocks. Subsequently, the isolated intermediates are subjected to flexible and diverse chemical modifications and functional group transformations *in vitro*. This strategy significantly reduces systemic complexity and avoids the challenges of catalyst mutual inhibition and reaction condition incompatibility, providing a pragmatic pathway for the commercial production of many high-value compounds (El-malek and Steinbüchel, 2022).

Modern research continues to deepen this strategy, shifting the focus from simple sequential operations toward the precise design of “interface chemistry” and the enhancement of process integration. In the biosynthetic part, constructing efficient “living factories” is fundamental. For instance, in the pharmaceutical industry, the directed evolution of *α*-ketoglutarate dependent dioxygenases (α-KGDs) can replace multiple chemical synthesis steps, enabling the highly selective and direct synthesis of key chiral intermediates for drugs such as Belzutifan (Reisenbauer et al., 2024). Furthermore, biocatalysts like imine reductases (IREDs) and reductive aminases (RedAms) have been successfully applied to the multi-ton scale synthesis of chiral amines due to their high stability and selectivity (Reisenbauer et al., 2024; Yao Z. et al., 2025). These engineered biomanufacturing platforms provide a rich and reliable feedstock library for subsequent chemical modifications.

One of the most representative chemical modification exemplars is dynamic kinetic resolution (DKR). A typical process involves first obtaining racemic intermediates such as alcohols or amines via fermentation or biocatalysis. After extraction, a metal racemization catalyst and an immobilized lipase are ingeniously combined in a chemical reactor (Kim et al., 2013). The metal catalyst is responsible for rapid substrate racemization to overcome thermodynamic yield limitations, while the biocatalyst enables directional conversion based on its exceptional enantioselectivity. In theory, their synergy allows for the production of optically pure compounds with 100% yield (Zhang J. et al., 2025). To overcome the bottleneck of traditional DKR systems, where high-temperature requirements limit enzyme selection, recent research has introduced novel catalysts such as cationic ruthenium indenylidene complexes. These catalysts exhibit excellent racemization activity even at room temperature, thereby greatly expanding the applicability and practicality of this synergistic strategy (Verho and Bäckvall, 2015).

Cutting-edge industrial case studies demonstrate the formidable power of this strategy in synthesizing complex drug molecules. For Molnupiravir, researchers at Merck & Co. developed an engineered ribosyl-1-kinase route that uses ribose as the starting material and directs a concise biocatalytic sequence toward the antiviral nucleoside scaffold. Compared with the initial route, the revised synthesis was 70% shorter and approximately seven-fold higher yielding, minimizing unnecessary isolation and protecting-group operations (McIntosh et al., 2021). Similarly, for the anti-HIV drug Islatravir, Merck designed a biocatalytic cascade that leverages a biomimetic nucleoside salvage pathway and five engineered enzymes, enabling efficient asymmetric synthesis without the protecting-group burden typical of purely chemical routes (Huffman et al., 2019). These cases illustrate the essence of post-fermentation or post-biosynthetic modification: biology or isolated enzymes efficiently and precisely construct chiral cores that are challenging to synthesize chemically, while subsequent functional group conversions or derivatizations can be accomplished by efficient chemical steps. Similar strategies with high degrees of biocatalytic-chemical integration have also been applied to the production of molecules like artemisinin and inositol (Giri et al., 2024; Siedentop and Rosenthal, 2022).

In semisynthetic artemisinin production, engineered *Saccharomyces cerevisiae* produces artemisinic acid at high level, and the isolated intermediate is converted chemically to artemisinin through oxygenation and rearrangement chemistry. This case illustrates a mature interface-molecule strategy: biology solves the supply problem for a plant-derived sesquiterpene precursor, while chemistry installs the endoperoxide pharmacophore that is difficult to manage *in vivo* (Kung et al., 2018; Paddon et al., 2013). Paclitaxel represents a complementary model. Because the taxane core is highly oxygenated and stereochemically dense, industrial routes have relied on renewable Taxus-derived or cell-culture-derived taxane intermediates and chemical finishing steps rather than full *de novo* chemical synthesis. Recent synthetic-biology and plant-cell-culture work highlights the same bottleneck: microorganisms can increasingly access taxadiene or partially oxidized taxanes, but complete biosynthesis and scalable late-stage oxygenation remain limiting, making semi-synthesis and post-biosynthetic modification essential (Yin et al., 2025).

Although current biological and chemical steps are strictly sequential, frontier explorations are actively working to blur the boundaries between the biological and chemical units (Pyser et al., 2021). For instance, *in situ* extractive fermentation techniques utilize adsorbent resins or biphasic systems to continuously remove and initially convert products during fermentation, alleviating product inhibition and improving overall efficiency. Concurrently, engineering microbial hosts to enhance the secretory output of target intermediates is also an important direction for simplifying subsequent chemical processing steps (Pyser et al., 2021). Of course, the inherent limitations of this strategy cannot be overlooked, particularly the material loss and cost increase from multiple separation and purification steps, as well as the room for improvement in overall atom economy. Therefore, it is best suited for scenarios where the biosynthetic pathway is complex but yields a “key platform molecule” from which a diverse range of products can be derived through a few highly efficient chemical transformations.

In summary, chemically enabled post-fermentation modification serves as a solid bridge translating the concept of bio-chemical synergy into practical application. It is not only a practical solution for the semi-synthesis of complex natural products like paclitaxel but has also demonstrated immense value in the rapid manufacturing of drugs to address global public health challenges. This strategy accumulates critical experience in molecular interface design and process integration, paving the way for more deeply integrated systems such as one-pot chemoenzymatic tandems and cell-free manufacturing platforms.

### 4.3 Chemoenzymatic methods

Chemoenzymatic catalysis represents the most core and advanced paradigm within the bio-chemical synergy strategy. It transcends simple sequential tandems by aiming to achieve spatiotemporal coordination and functional complementarity between abiotic catalysts, namely chemical catalysts, and biological catalysts, namely enzymes, within a single reaction vessel. The ultimate goal of this “one-pot” synergy is to create a reaction system that combines the broad scope of chemical transformations with the high selectivity and efficiency of enzymatic catalysis, thereby accomplishing complex syntheses that are difficult to realize using any single catalytic mode alone (Reisenbauer et al., 2024).

Catalyst compatibility should be evaluated along at least four axes. The first is pH: most enzymes and whole cells perform best near neutral pH, whereas acid-promoted cyclizations, iminium chemistry, and base-promoted couplings may require strongly acidic or basic media. The second is temperature: many metal-catalyzed racemizations, hydrogenations, and C-H activations become efficient only above 60–80 °C, while most mesophilic enzymes lose activity or structural integrity in this range. The third is water activity and solvent: enzymes require an aqueous hydration shell, but organometallic catalysts and moisture-sensitive reagents may require anhydrous or low-water media. The fourth is gas and redox environment: oxygen is essential for many oxidases and P450 systems, inhibitory to some radical or low-valent metal catalysts, and also modulates photocatalytic quenching. Therefore, compatibility is not a binary property but a design space involving solvent, water content, pH, temperature, oxygen transfer, redox buffering, and residence time (Liao et al., 2019; Park et al., 2025; Verho and Bäckvall, 2015; Wang et al., 2024).

Practical solutions include sequential addition, immobilization of one or both catalysts, protein or polymer shells around metal centers, biphasic media that partition toxic reagents away from enzymes, room-temperature racemization catalysts for DKR, flow reactors with separate temperature or oxygen zones, and cell-free systems in which catalyst concentrations can be tuned without maintaining cell viability (Tang et al., 2024; Verho and Bäckvall, 2015; Wang et al., 2024). These strategies explain why cell-free and flow platforms are especially attractive for biochemical integration: they retain the selectivity of enzymes while allowing tighter control of mass transfer, light irradiation, oxygen supply, and catalyst contact time.

**Figure 7:**
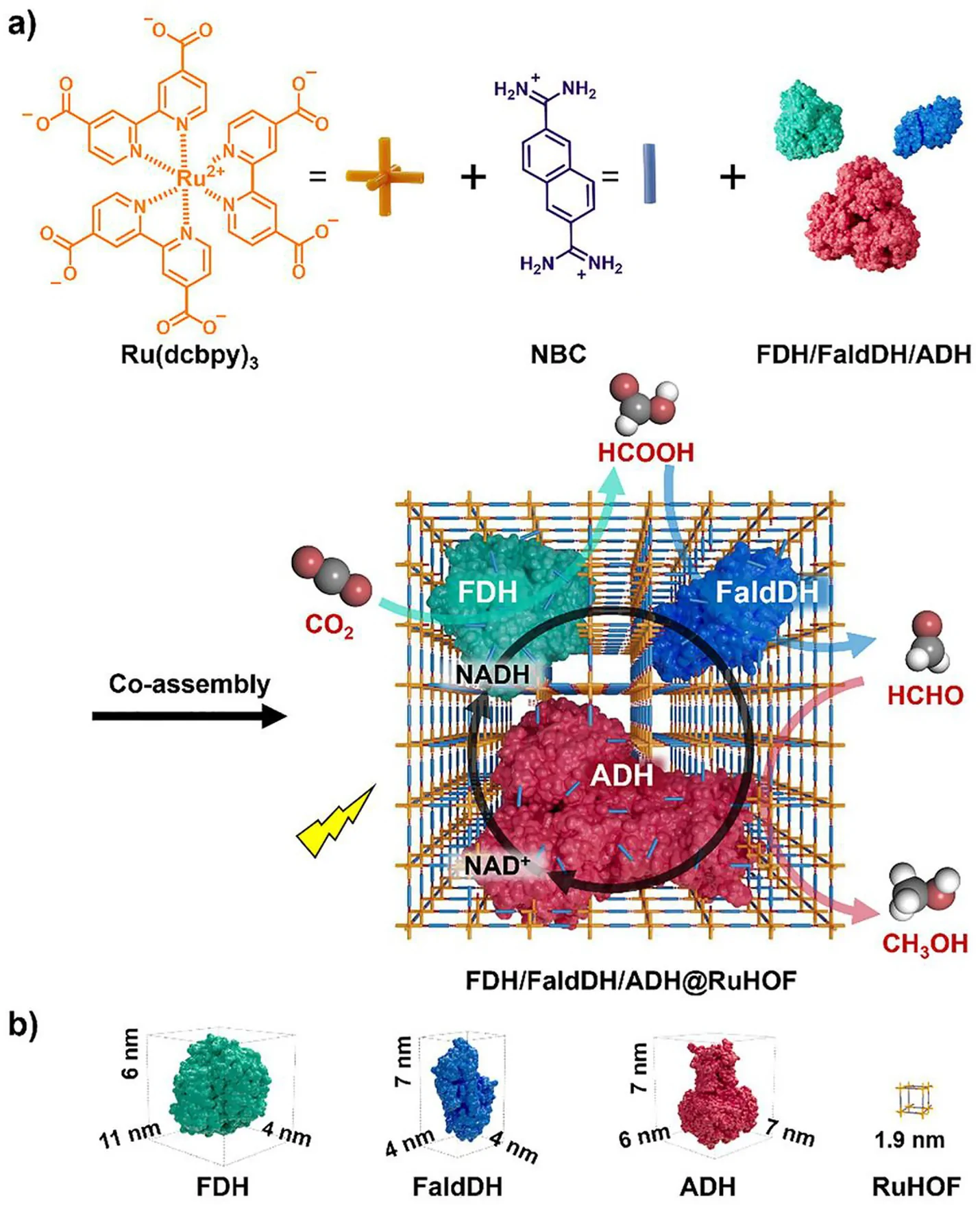
**(a)** Schematic illustration of the *in situ* co-assembly of FDH, FaldDH, and ADH with precursors of photoactive HOFs to construct a photo-enzymatic tandem system, FDH/FaldDH/ADH@RuHOF, for NADH-mediated CO_2_ tandem reduction to produce methanol. **(b)** The dimensions of the enzymes of FDH, FaldDH, ADH, and RuHOF. Source: Reproduced from Tang et al. (2025) with permission from Wiley.

The key to achieving deep synergy lies in resolving catalyst compatibility issues, and cutting-edge research is making breakthroughs through strategies of “compartmentalization” and “biomimetic assembly.” As shown in Figure 7, inspired by the natural proteinaceous organelle, the carboxysome of cyanobacteria, Tian et al. constructed an artificial photosynthetic system. The researchers successfully co-assembled, via hydrogen bonding in an aqueous phase, a ruthenium bipyridine photocatalyst center with three enzymes—formate dehydrogenase, formaldehyde dehydrogenase, and alcohol dehydrogenase—into an organized microenvironment in a one-pot manner (Tang et al., 2025). This system perfectly integrates photocatalysis with a multi-enzyme tandem, achieving the efficient and continuous conversion of carbon dioxide to methanol, with performance and stability far surpassing those of a simple mixture of the catalysts (Tang et al., 2025). This study demonstrates that spatially organizing catalysts through precise supramolecular assembly can not only physically bring incompatible catalytic sites into proximity to accelerate intermediate transfer but also provide an optimal microenvironment for each. This approach serves as a powerful tool for realizing highly efficient chemoenzymatic synergy.

In the field of total synthesis of complex natural products, the chemoenzymatic strategy is demonstrating transformative power. For instance, targeting bisbenzylisoquinoline alkaloids with significant biological activities, Lei et al. (2024) developed an efficient convergent chemoenzymatic synthesis route. The core of this strategy is the use of norcoclaurine synthase (NCS) to catalyze a highly enantioselective Pictet–Spengler reaction, efficiently constructing a key chiral fragment. This is then seamlessly connected to chemical steps, such as an intramolecular Ullmann coupling reaction, to complete the final skeletal assembly (Lei et al., 2024). This approach bypasses the lengthy steps and cumbersome protecting group manipulations common in traditional total synthesis. It seamlessly combines the exceptional efficiency of enzymes in constructing specific stereogenic centers with the flexibility of chemical synthesis in forming carbon–carbon and carbon-heteroatom bonds, providing a practical pathway to access these scarce natural drug molecules and their analogs (Dong et al., 2024; Lei et al., 2024).

Recent examples broaden this concept beyond a single reactor. Photobiocatalytic alkene isomerization/reduction uses a photocatalyst to equilibrate E/Z alkene mixtures while an ene-reductase selectively reduces the reactive isomer, converting a stereoselective enzyme into a stereoconvergent system (Litman et al., 2018). Flow biocatalysis adds an engineering solution: immobilized enzymes and packed-bed or segmented-flow reactors enhance heat and mass transfer, enable catalyst reuse, and offer separate residence-time control for enzymatic, metal-catalyzed, photo-, or electrochemical modules (Tang et al., 2024). Biofoundry-enabled synthesis closes the loop upstream by accelerating construction and testing of strains, enzymes, and genetic circuits through automated DBTL workflows, making it possible to identify interface molecules and catalyst-compatible variants more rapidly (Yu C. Y. et al., 2025).

Furthermore, purely enzymatic “one-pot” multienzyme tandem systems themselves constitute a crucial foundation of chemoenzymatic catalysis. By designing and reconstructing natural metabolic pathways, they enable the direct conversion of simple substrates into high-value chemicals (Yao et al., 2024). For example, a recent study designed an ingenious multienzyme tandem capable of efficiently synthesizing L-threitol from the single C1 compound formaldehyde. This process relies on the sequential catalysis of formolase, fructose-6-phosphate aldolase, and a newly discovered L-threitol dehydrogenase (Gong et al., 2025). The system also integrates a self-sustained NADH regeneration cycle, ensuring catalytic economy, and achieved a product concentration as high as 49.6 g/L in a single reactor, fully demonstrating the potential of modular enzyme tandems in green synthesis (Gong et al., 2025). Although such systems do not incorporate traditional chemical catalysts, their design philosophy of integrating multiple catalytic functions, including cofactor regeneration, in a “one-pot” manner is highly consistent with chemoenzymatic catalysis and provides a potential interface for introducing non-natural chemical catalytic steps.

In summary, chemoenzymatic catalysis is reshaping the logic of complex molecule synthesis by integrating the powerful capabilities of chemical catalysis with the precise control of enzymatic catalysis. Whether constructing hybrid systems through biomimetic assembly or intelligently switching between biological and chemical modules within a synthetic route, the core pursuit is the optimal convergence of synthetic pathways and the maximization of atom economy. Although challenges remain regarding catalyst compatibility and reaction condition homogenization, the increasing integration of protein engineering, synthetic biology, and materials science positions chemoenzymatic catalysis as a key engine driving green pharmaceutical manufacturing and sustainable chemical production (Reisenbauer et al., 2024).

### 4.4 Cell-free biosynthesis-chemical integration systems

**Figure 8:**
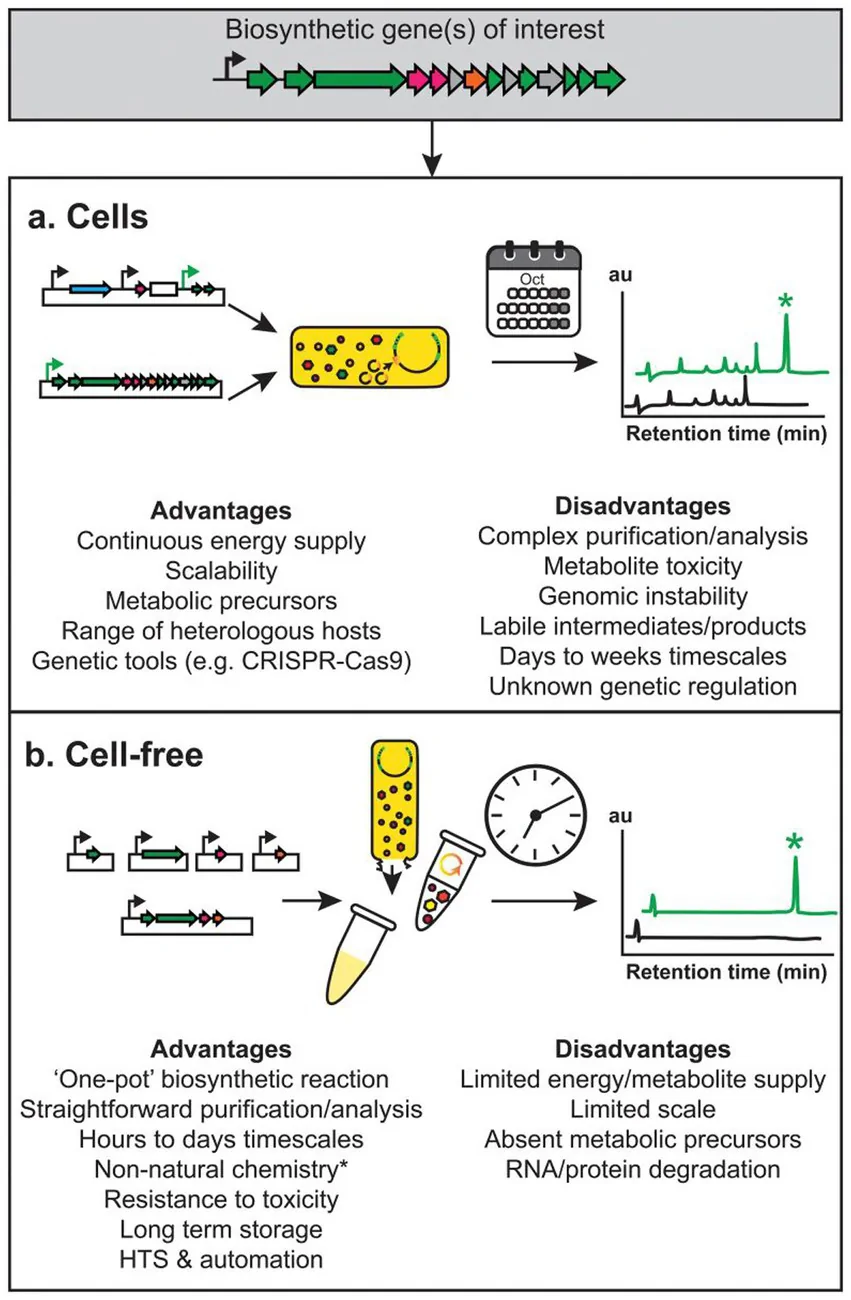
Overview of the advantages and disadvantages of **(a)** whole cell and **(b)** cell-free approaches to natural product biosynthesis and discovery. Source: Reproduced from Rice et al. (2025), licensed under CC BY 3.0.

Cell-free biosynthetic-chemical integrated systems represent the most controllable and design-flexible frontier paradigm within the bio-chemical synergy strategy. This approach completely removes the physical barrier of the cell membrane and the physiological constraints of cell growth, “extracting” complex biosynthetic machinery to construct a highly engineered “bioreactor” *in vitro*. This open system allows researchers to precisely mix purified enzymes, cofactors, chemical catalysts, and unnatural substrates at any desired concentration and ratio, thereby enabling the rational reconstruction and seamless integration of complex metabolic pathways (Kelwick et al., 2020). As shown in Figure 8, its core advantage lies in fully leveraging the “bottom-up” modular engineering capabilities of synthetic biology, providing an ideal platform for rapid prototyping, the synthesis of toxic molecules, and, in particular, deep coupling with synthetic chemical reactions (Rice et al., 2025).

Cell-free systems are especially advantageous for bio-chemical integration because they decouple catalytic performance from cell viability. Metal ions, organic cosolvents, light, unnatural cofactors, or toxic intermediates can be introduced at concentrations that would inhibit growth, while enzymes and cofactors can be replenished, removed, or immobilized as needed. Unlike whole-cell systems, an open cell-free reactor allows direct control of enzyme ratios, cofactor regeneration, oxygen transfer, pH, ionic strength, and the timing of chemical catalyst addition; however, the absence of cellular homeostasis also means that energy supply, cofactor stability, protease/RNase activity, and cost of enzyme or lysate preparation become major constraints (Kelwick et al., 2020; Rice et al., 2025).

In the field of natural product discovery and synthesis, cell-free systems are demonstrating significant potential. Traditional heterologous expression based on living cells is often limited by issues such as substrate/product toxicity, insufficient precursor supply, or the difficulty in activating “silent” biosynthetic gene clusters (Rice et al., 2025). Cell-free systems, however, can directly bypass these obstacles. For example, Bailey et al. utilized cell extracts from natural product producers like *Streptomyces* to rapidly activate and characterize their entire biosynthetic gene clusters *in vitro*, facilitating the discovery of new antibiotics or the elucidation of assembly logic for complex polyketides and nonribosomal peptides (Rice et al., 2025). Furthermore, through carefully designed multi-enzyme tandem reactions, efficient one-pot conversion from simple substrates to high-value chemicals can be achieved. Gao et al. designed a tandem system requiring only five enzymes and no expensive cofactors to efficiently convert glucose into pyruvate, an important platform chemical, highlighting the outstanding capability of cell-free systems in pathway simplification and economic viability (Tan et al., 2023). This “plug-and-play” characteristic makes it exceptionally convenient to replace, optimize, or introduce non-natural enzymes within a metabolic pathway (Kelwick et al., 2020).

The deep integration of cell-free systems with synthetic chemistry is key to advancing toward a higher level of synergy. In an open reaction environment, abiotic chemical catalytic steps can be directly introduced into enzymatic tandems. For instance, after an enzymatic synthesis yields a chiral intermediate, compatible metal catalysts can be added directly into the same reaction mixture without isolation or purification to perform subsequent transformations such as C–H bond functionalization, cross-coupling, or photocatalytic cyclization. This enables the creation of “unnatural” natural product analogs that do not exist in nature. This integration overcomes the tolerance limits of living cells toward organic solvents, metal ions, or toxic intermediates. More importantly, cell-free transcription-translation systems can synthesize specific functional enzymes on-demand within the reaction vessel. This means that by adding exogenous DNA templates and chemical inducers, the composition and flux of the biosynthetic pathway can be dynamically regulated, achieving programmed spatiotemporal synergy between biocatalysis and chemocatalysis (Kelwick et al., 2020; Rice et al., 2025).

For integrated natural-product diversification, the most useful cell-free workflow is therefore a programmable sequence in which a biosynthetic enzyme set first generates a reactive interface molecule, followed by *in situ* addition of a compatible metal, organo-, photo-, or electrochemical catalyst. If incompatibility remains severe, the same open platform can implement temporal separation by filtration, immobilized enzyme modules, resin capture, or flow-through catalyst cartridges. In this way, cell-free systems occupy a middle ground between whole-cell fermentation and classical organic synthesis: they are less self-sustaining than living cells, but far more chemically permissive and analytically transparent.

In summary, cell-free biosynthetic-chemical integrated systems serve not only as powerful production tools but also as fundamental research platforms for exploring the interface between biological and chemical catalysis and for reconstructing life processes. They liberate metabolic engineering from the cellular “black box,” enabling precise, atom-level manipulation of material transformation pathways. Although challenges remain in terms of system stability and scale-up costs, they have an attractive path toward rapidly diversifying complex molecular structures, developing novel bio-chemical hybrid reactions, and ultimately realizing on-demand, customized molecular manufacturing (Rice et al., 2025).

## 5 Conclusion

This review explores synergistic strategies integrating synthetic biology’s microbial living factories with one-pot chemical tandem catalysis for the biosynthesis and diversification of natural products. The central rationale is a division of labor: biological systems provide stereoselective, convergent construction of complex scaffolds from renewable feedstocks, whereas chemical, chemoenzymatic, and cell-free systems provide divergent modification modes that expand structural space beyond the limits of native metabolism. In the near term, the most realistic industrial routes will involve rational choice among sequential post-fermentation modification, mild one-pot chemoenzymatic coupling, and open cell-free integration rather than forcing all biological and chemical steps into a single living cell.

Several bottlenecks define the next stage of the field. Catalyst compatibility remains the central molecular challenge because pH, temperature, water activity, oxygen demand, and redox state must be simultaneously compatible with enzymes, cells, and abiotic catalysts. Scale-up remains the central engineering challenge because oxygen transfer, mixing time, substrate gradients, product toxicity, and genetic stability can convert a high-performing laboratory strain into a low-yielding industrial process. Downstream purification and catalyst recovery also require greater attention, especially when residual metals, enzyme impurities, DNA/RNA fragments, or non-natural analogs must meet pharmaceutical quality requirements.

A practical research roadmap can be organized into five steps. First, the target natural-product class should be matched to its dominant biosynthetic bottleneck, such as P450 oxygenation in terpenoids, domain communication in PKS/NRPS systems, or glycosyl-donor balance in phenolics. Second, an interface molecule with both high biosynthetic accessibility and selective chemical handles should be defined. Third, biocompatible catalysts and reaction sequences should be screened under realistic aqueous, oxygen, temperature, and impurity conditions. Fourth, the process should be validated in scale-down, flow, or cell-free platforms that reproduce industrial mass-transfer and residence-time constraints. Fifth, AI-assisted and biofoundry-enabled DBTL cycles should be used to optimize strain genotype, enzyme variants, catalyst loading, solvent composition, and separation strategy against quantitative metrics, including titer, rate, yield, selectivity, E-factor, and cost of goods.

Regulatory and translational considerations should be incorporated from the beginning. Engineered organisms require containment and genetic-stability assessment; chemically modified natural-product analogs require impurity profiling, residual-catalyst control, and reproducible GMP-compatible workflows; and sustainable manufacturing claims require life-cycle and solvent/catalyst-recycling analyses rather than isolated yield data alone. Standardized reporting of fermentation scale, dissolved-oxygen control, catalyst concentration, solvent composition, residence time, downstream recovery, and product purity will make future studies more comparable and more useful for industrial adoption.

Overall, bio-chemical integration is more than a production technology. It is an engineering philosophy that connects biological convergence with chemical divergence, allowing researchers to design natural-product-like molecules from genetic information, renewable feedstocks, and programmable catalytic modules. With advances in compatible catalysis, scale-aware process design, artificial metalloenzymes, flow systems, cell-free platforms, and automated biofoundries, this field is positioned to accelerate green pharmaceutical manufacturing and natural-product-based drug discovery.

## References

[ref1] Arulrajah P. Lievonen A. E. Subaşı D. Pagal S. Weuster-Botz D. Heins A.-L. (2025). Scale-down bioreactors—comparative analysis of configurations. Bioprocess Biosyst. Eng. 48, 1619–1635. doi: 10.1007/s00449-025-03182-w, 40471288 PMC12460589

[ref2] Authors (2025). One reactor, two reactor. Nat. Chem. Eng. 2, 665–665. doi: 10.1038/s44286-025-00323-6

[ref3] Bai L. Jiang X. (2023). Catalytic domino reaction: a promising and economic tool in organic synthesis. Chem. Catal. 3:100752. doi: 10.1016/j.checat.2023.100752

[ref4] Bondžić B. P. (2015). Rh catalyzed multicomponent tandem and one-pot reactions under hydroformylation conditions. J. Mol. Catal. A Chem. 408, 310–334. doi: 10.1016/j.molcata.2015.07.026

[ref5] Boyle P. M. Silver P. A. (2012). Parts plus pipes: synthetic biology approaches to metabolic engineering. Metab. Eng. 14, 223–232. doi: 10.1016/j.ymben.2011.10.003, 22037345 PMC3293987

[ref6] Cao Y. Li J. Liu L. Du G. Liu Y. (2024). Harnessing microbial heterogeneity for improved biosynthesis fueled by synthetic biology. Synth. Syst. Biotechnol. 10, 281–293. doi: 10.1016/j.synbio.2024.11.004, 39686977 PMC11646789

[ref7] Chen Z. Aota Y. Nguyen H. M. H. Dong V. M. (2019). Dynamic kinetic resolution of aldehydes by hydroacylation. Angew. Chem. Int. Ed. 58, 4705–4709. doi: 10.1002/anie.201900545, 30740841 PMC6501835

[ref8] Chen M. Liu J. Duan P. Li M. Liu W. (2016). Biosynthesis and molecular engineering of templated natural products. Natl. Sci. Rev. 4, 553–575. doi: 10.1093/nsr/nww045

[ref9] Chen G. Liu J.-M. Ruan L.-X. Shi S.-L. (2025). Selective dynamic kinetic asymmetric aldehyde–alkyne reductive coupling. Nat. Synth. 4, 1630–1639. doi: 10.1038/s44160-025-00887-4

[ref10] Crater J. S. Lievense J. C. (2018). Scale-up of industrial microbial processes. FEMS Microbiol. Lett. 365:fny138. doi: 10.1093/femsle/fny138, 29860483 PMC5995164

[ref11] de Frias U. A. Pereira G. K. B. Guazzaroni M.-E. Silva-Rocha R. (2018). Boosting secondary metabolite production and discovery through the engineering of novel microbial biosensors. Biomed. Res. Int. 2018, 1–11. doi: 10.1155/2018/7021826, 30079350 PMC6069586

[ref12] Dong H. Guo N. Hu D. Hong B. Liao D. Zhu H. J. . (2024). Chemoenzymatic total synthesis of alchivemycin a. Nat. Synth. 3, 1124–1133. doi: 10.1038/s44160-024-00577-7

[ref13] Du Y. H. Kornecki M. Hoffmann M. Neubauer P. Junne S. (2022). Optimization and scale-up of fermentation processes driven by models. Bioengineering 9:473. doi: 10.3390/bioengineering9090473 36135019 PMC9495923

[ref14] El-malek F. A. Steinbüchel A. (2022). Post-synthetic enzymatic and chemical modifications for novel sustainable polyesters. Front. Bioeng. Biotechnol. 9:817023. doi: 10.3389/fbioe.2021.817023, 35071219 PMC8766639

[ref15] Fogg D. E. dos Santos E. N. (2004). Tandem catalysis: a taxonomy and illustrative review. Coord. Chem. Rev. 248, 2365–2379. doi: 10.1016/j.ccr.2004.05.012

[ref16] Ford T. J. Silver P. A. (2015). Synthetic biology expands chemical control of microorganisms. Curr. Opin. Chem. Biol. 28, 20–28. doi: 10.1016/j.cbpa.2015.05.012, 26056951 PMC4961477

[ref17] García-Ochoa F. Gómez E. (2009). Bioreactor scale-up and oxygen transfer rate in microbial processes: an overview. Biotechnol. Adv. 27, 153–176. doi: 10.1016/j.biotechadv.2008.10.006, 19041387

[ref18] Giaveri S. Bohra N. Diehl C. Yang H. Y. Ballinger M. Paczia N. . (2024). Integrated translation and metabolism in a partially self-synthesizing biochemical network. Science 385, 174–178. doi: 10.1126/science.adn3856, 38991083

[ref19] Giri P. Lim S. Khobragade T. P. Pagar A. D. Patil M. D. Sarak S. . (2024). Biocatalysis enables the scalable conversion of biobased furans into various furfurylamines. Nat. Commun. 15:6371. doi: 10.1038/s41467-024-50637-x 39075048 PMC11286754

[ref20] Gong S. Li T. Tang Z. Tan Z. Zhang R. Olsen K. . (2025). One-pot enzymatic synthesis of L-threitol from C1 formaldehyde. Green Chem. 27, 2189–2196. doi: 10.1039/d4gc05638h

[ref21] He F. Murabito E. Westerhoff H. V. (2016). Synthetic biology and regulatory networks: where metabolic systems biology meets control engineering. J. R. Soc. Interface 13:20151046. doi: 10.1098/rsif.2015.1046, 27075000 PMC4874428

[ref22] Hinzpeter F. Gerland U. Tostevin F. (2017). Optimal compartmentalization strategies for metabolic microcompartments. Biophys. J. 112, 767–779. doi: 10.1016/j.bpj.2016.11.3194, 28256236 PMC5340097

[ref23] Hsu H.-T. Trantow B. M. Waymouth R. M. Wender P. A. (2015). Bioorthogonal catalysis: a general method to evaluate metal-catalyzed reactions in real time in living systems using a cellular luciferase reporter system. Bioconjug. Chem. 27, 376–382. doi: 10.1021/acs.bioconjchem.5b00469, 26367192 PMC4772775

[ref24] Huang J. Liu Z. Bloomer B. J. Clark D. S. Mukhopadhyay A. Keasling J. D. . (2021). Unnatural biosynthesis by an engineered microorganism with Heterologously expressed natural enzymes and an artificial metalloenzyme. Nat. Chem. 13, 1186–1191. doi: 10.1038/s41557-021-00801-3, 34650235 PMC8879416

[ref25] Huang W. Wang J. Tang G. (2011). Synthetic biology toward medicinal natural products. Chin. Bull. Life Sci. 23, 891–899.

[ref26] Huang Z. Xie S. Liu R.-Z. Xiang C. Yao S. Zhang L. (2025). Plug-and-play engineering of modular polyketide synthases. Nat. Chem. Biol. 21, 1361–1367. doi: 10.1038/s41589-025-01878-4, 40251436

[ref27] Huang C. Zhao J. Tan Z. Dai S. Wang B. Xie Z. . (2025). BioFuse: a programmable timer switch of gene expression. Sci. Adv. 11:eadv7892. doi: 10.1126/sciadv.adv7892, 40864720 PMC12383275

[ref28] Huffman M. A. Fryszkowska A. Alvizo O. Borra-Garske M. Campos K. R. Canada K. A. . (2019). Design of an in vitro biocatalytic Cascade for the manufacture of Islatravir. Science 366, 1255–1259. doi: 10.1126/science.aay8484, 31806816

[ref29] Ishikawa H. Suzuki T. Hayashi Y. (2009). High-yielding synthesis of the anti-influenza Neuramidase inhibitor (−)-oseltamivir by three “one-pot” operations. Angew. Chem. Int. Ed. 48, 1304–1307. doi: 10.1002/anie.200804883, 19123206

[ref30] Ke J. Yoshikuni Y. (2020). Multi-chassis engineering for heterologous production of microbial natural products. Curr. Opin. Biotechnol. 62, 88–97. doi: 10.1016/j.copbio.2019.09.005, 31639618

[ref31] Kelwick R. J. R. Webb A. J. Freemont P. S. (2020). Biological materials: the next frontier for cell-free synthetic biology. Front. Bioeng. Biotechnol. 8:399. doi: 10.3389/fbioe.2020.00399, 32478045 PMC7235315

[ref32] Kim C. Lee J. Cho J. Oh Y. Choi Y. K. Choi E. . (2013). Kinetic and dynamic kinetic resolution of secondary alcohols with ionic-surfactant-coated *Burkholderia cepacia* lipase: substrate scope and enantioselectivity. J. Org. Chem. 78, 2571–2578. doi: 10.1021/jo3027627, 23406287

[ref33] Kim G. Lee D. Kim J. H. Kim S. D. Kim H. Kim J. H. . (2025). Engineering modular enzyme assembly: synthetic Interface strategies for natural products biosynthesis applications. Nat. Prod. Rep. 42, 1489–1506. doi: 10.1039/d5np00027k, 40576373

[ref34] Kretschmer S. Kortemme T. (2022). Advances in the computational Design of Small-Molecule-Controlled Protein-Based Circuits for synthetic biology. Proc. IEEE 110, 659–674. doi: 10.1109/jproc.2022.3157898, 36531560 PMC9754107

[ref35] Kung S. H. Lund S. Murarka A. McPhee D. Paddon C. J. (2018). Approaches and recent developments for the commercial production of semi-synthetic artemisinin. Front. Plant Sci. 9:87. doi: 10.3389/fpls.2018.00087, 29445390 PMC5797932

[ref36] Lei X. Wang J. Zhao J. Yu Z. Wang S. Guo F. . (2024). Concise and modular chemoenzymatic total synthesis of bisbenzylisoquinoline alkaloids. Angew. Chem. Int. Ed. 64:e202414340. doi: 10.1002/anie.202414340 39305151

[ref37] Liao X. Jiang Y. Lai S. Liu Y. Wang S. Xiong X. (2019). Chemoenzymatic relay reaction and its applications in highly efficient and green synthesis of high-value chiral compounds. Chin. J. Org. Chem. 39, 668–668. doi: 10.6023/cjoc201807038

[ref38] Litman Z. C. Wang Y. Zhao H. Hartwig J. F. (2018). Cooperative asymmetric reactions combining photocatalysis and enzymatic catalysis. Nature 560, 355–359. doi: 10.1038/s41586-018-0413-7, 30111790

[ref39] Liu F. He H. Li Y. Zhang X. Xu X. Wang L. (2023). Artificial small molecules as cofactors and biomacromolecular building blocks in synthetic biology: design, synthesis, applications, and challenges. Molecules 28:5850. doi: 10.3390/molecules28155850 37570818 PMC10421094

[ref40] Liu J. Wang X. Z. Dai G. Zhang Y.-M. Bian X. (2022). Microbial chassis engineering drives heterologous production of complex secondary metabolites. Biotechnol. Adv. 59, 107966–107966. doi: 10.1016/j.biotechadv.2022.107966, 35487394

[ref41] Ma X. Zhang W. (2022). Recent developments in one-pot stepwise synthesis (OPSS) of small molecules. iScience 25, 105005–105005. doi: 10.1016/j.isci.2022.105005, 36093043 PMC9460791

[ref42] McIntosh J. A. Benkovics T. Silverman S. M. Huffman M. A. Kong J. Maligres P. E. . (2021). Engineered Ribosyl-1-kinase enables concise synthesis of Molnupiravir, an antiviral for COVID-19. ACS Central Sci. 7, 1980–1985. doi: 10.1021/acscentsci.1c00608, 34963891 PMC8704035

[ref43] Nicolaou K. C. Chen J. S. (2009). The art of Total synthesis through Cascade reactions. Chem. Soc. Rev. 38, 2993–3009. doi: 10.1039/b903290h, 19847336 PMC2766600

[ref44] Olughu W. Nienow A. W. Hewitt C. J. Rielly C. D. (2020). Scale-down studies for the scale-up of a recombinant *Corynebacterium glutamicum* fed-batch fermentation: loss of homogeneity leads to lower levels of cadaverine production. J. Chem. Technol. Biotechnol. 95, 675–685. doi: 10.1002/jctb.6248, 32139953 PMC7043379

[ref45] Paddon C. J. Westfall P. J. Pitera D. J. Benjamin K. Fisher K. McPhee D. . (2013). High-level semi-synthetic production of the potent antimalarial artemisinin. Nature 496, 528–532. doi: 10.1038/nature12051, 23575629

[ref46] Park J. Kim M.-J. Choi J. H. Ahn Y.. (2025). CN1622932A - resolution of chiral compounds using aminocyclopentadienyl ruthenium catalysts - Google patents. Available online at: https://patents.google.com/patent/CN1622932A/en (Accessed December 18, 2025)

[ref47] Peng Y.-J. Chen Y. Zhou C.-Z. Miao W. Jiang Y.-L. Zeng X. . (2024). Modular catalytic activity of nonribosomal peptide synthetases depends on the dynamic interaction between adenylation and condensation domains. Structure 32, 440–452.e4. doi: 10.1016/j.str.2024.01.010, 38340732

[ref48] Pyser J. B. Chakrabarty S. Romero E. O. Narayan A. R. H. (2021). State-of-the-art biocatalysis. ACS Central Sci. 7, 1105–1116. doi: 10.1021/acscentsci.1c00273, 34345663 PMC8323117

[ref49] Ray K. A. Saif N. Keatinge-Clay A. T. (2024). Modular polyketide synthase Ketosynthases collaborate with upstream dehydratases to install double bonds. Chem. Commun. 60, 8712–8715. doi: 10.1039/d4cc03034f, 39056119 PMC11321453

[ref50] Reisenbauer J. C. Sicinski K. M. Arnold F. H. (2024). Catalyzing the future: recent advances in chemical synthesis using enzymes. Curr. Opin. Chem. Biol. 83, 102536–102536. doi: 10.1016/j.cbpa.2024.102536, 39369557 PMC11588546

[ref51] Renda B. A. Hammerling M. J. Barrick J. E. (2014). Engineering reduced evolutionary potential for synthetic biology. Mol. BioSyst. 10, 1668–1678. doi: 10.1039/c3mb70606k, 24556867 PMC4043932

[ref52] Rice A. J. Sword T. T. Chengan K. Mitchell D. A. Mouncey N. J. Moore S. J. . (2025). Cell-free synthetic biology for natural product biosynthesis and discovery. Chem. Soc. Rev. 54, 4314–4352. doi: 10.1039/d4cs01198h, 40104998 PMC11920963

[ref53] Sagharyan M. Mohammadbagherlou S. Samari E. Zargar M. Ghorbani A. Chen M. (2025). Synthetic biology and metabolic engineering strategies in identifying and producing plant natural products; with emphasis on the CRISPR/Cas systems. Ind. Crop. Prod. 230:121060. doi: 10.1016/j.indcrop.2025.121060

[ref54] Siedentop R. Rosenthal K. (2022). Industrially relevant enzyme cascades for drug synthesis and their ecological assessment. Int. J. Mol. Sci. 23:3605. doi: 10.3390/ijms23073605, 35408960 PMC8998672

[ref55] Sun T. Huang M. Liu B. Chen X. (2025). Advances in microbial genome reduction and optimization based on synthetic biology. Sci. Technol. Food Ind. 46, 394–402. doi: 10.13386/j.issn1002-0306.2023120354

[ref56] Tan X. Guo S. Wang Y. Zhang Y. Zhang H. Lü C. . (2023). Multi-enzyme cascade excluding costly cofactors for pyruvate production from glucose. Chem. Eng. J. 463:142473. doi: 10.1016/j.cej.2023.142473

[ref57] Tang J. Lei Y. Tang Q. Lei S. Qi Q. Li Z. . (2025). Integration of multiple enzymes within hydrogen-bonded organic frameworks for efficient Cascade photocatalytic CO _2_ -to-methanol conversion in water. Angew. Chem. Int. Ed. 64, e202516599–e202516599. doi: 10.1002/anie.202516599, 40954535

[ref58] Tang Z. Oku Y. Matsuda T. (2024). Application of immobilized enzymes in flow biocatalysis for efficient synthesis. Org. Process. Res. Dev. 28, 1308–1326. doi: 10.1021/acs.oprd.3c00405

[ref59] Trinh C. T. Mendoza B. (2016). Modular cell design for rapid, efficient strain engineering toward industrialization of biology. Curr. Opin. Chem. Eng. 14, 18–25. doi: 10.1016/j.coche.2016.07.005

[ref60] Verho O. Bäckvall J.-E. (2015). Chemoenzymatic dynamic kinetic resolution: a powerful tool for the preparation of enantiomerically pure alcohols and amines. J. Am. Chem. Soc. 137, 3996–4009. doi: 10.1021/jacs.5b01031, 25730714 PMC4415027

[ref61] Wang J. Nielsen J. Liu Z. (2021). Synthetic biology advanced natural product discovery. Meta 11:785. doi: 10.3390/metabo11110785, 34822443 PMC8617713

[ref62] Wang K. Wang W. Lou D. Zhang J. Chi C. Bäckvall J.-E. . (2024). Overcoming the limitations of transition-metal catalysis in the chemoenzymatic dynamic kinetic resolution (DKR) of atropisomeric bisnaphthols. ACS Cent. Sci. 10, 2099–2110. doi: 10.1021/acscentsci.4c01370, 39634225 PMC11613327

[ref63] Wang Y. Zhou J. Wang F. Xin X. Chen Z. Tao Y. . (2025). Integrated cofactor-centric engineering and energy flux optimization enable high-efficient D-pantothenic acid production in *Escherichia coli*. Chem. Eng. J. 524:169179. doi: 10.1016/j.cej.2025.169179

[ref64] Xu W. Wei P. Chen L. Gao L. Xia X. (2025). Engineering Electron transfer flux between cytochrome P450 enzyme and P450 reductase to enhance serotonin production in *Escherichia coli*. Adv. Sci. 12, e14859–e14859. doi: 10.1002/advs.202414859, 40407227 PMC12376664

[ref65] Yan H. Li S. Wang W. (2025). Reprogramming naturally evolved switches for Streptomyces chassis development. Trends Biotechnol. 43, 12–15. doi: 10.1016/j.tibtech.2024.07.001, 39054219

[ref66] Yang D. Liang H. Li X. Zhang C. Lu Z. Ma X. (2025). Unleashing the potential of microbial biosynthesis of monoterpenes via enzyme and metabolic engineering. Biotechnol. Adv. 79:108525. doi: 10.1016/j.biotechadv.2025.108525, 39921018

[ref67] Yao Z. Meng R. Zhou Z. Yu L. Wu Z. Tang L. . (2025). Engineering an imine reductase for enantioselective synthesis of Atropisomeric amides. J. Am. Chem. Soc. 147, 40616–40625. doi: 10.1021/jacs.5c12724, 41140043

[ref68] Yao M. Wang H. Wang Z. Song C. Sa X. Du W. . (2024). Construct phenylethanoid glycosides harnessing biosynthetic networks, protein engineering and one-pot multienzyme cascades. Angew. Chem. Int. Ed. 63, e202402546–e202402546. doi: 10.1002/anie.202402546, 38616162

[ref69] Yao S. Xie S. Liu R.-Z. Huang Z. Zhang L. (2025). Expanding catalytic versatility of modular polyketide synthases for alcohol biosynthesis. Nat. Chem. Biol. 21, 1368–1375. doi: 10.1038/s41589-025-01883-7, 40251435

[ref70] Yin J.-Y. Lai M. Yu X.-Y. Su D.-D. Xiong X.-Y. Li Y.-L. (2025). Comprehensive strategies for paclitaxel production: insights from plant cell culture, endophytic microorganisms, and synthetic biology. Hortic. Res. 12:uhae346. doi: 10.1093/hr/uhae346, 40061810 PMC11890030

[ref71] Yook G. Nam J. Jo Y. Yoon H. Yang D. (2025). Metabolic engineering approaches for the biosynthesis of antibiotics. Microb. Cell Factories 24:35. doi: 10.1186/s12934-024-02628-2, 39891166 PMC11786382

[ref72] Yu C. Y. Ang G. Y. Mat Isa N. Chan K.-G. (2025). Frontiers in biofoundry: opportunities and challenges. Front. Synth. Biol. 3:1630026. doi: 10.3389/fsybi.2025.1630026

[ref73] Yu B. Huang Y. Zhang H. Huang H. (2025). Adaptive dynamic kinetic resolution enables alteration of chiral induction with ring sizes. Nat. Chem. 17, 1256–1264. doi: 10.1038/s41557-025-01850-8, 40550971

[ref74] Yu R. Wen W. (2011). Artemisinin biosynthesis and its regulatory enzymes: Progress and perspective. Pharmacogn. Rev. 5, 189–194. doi: 10.4103/0973-7847.91118, 22279377 PMC3263054

[ref75] Zhang J. Huo X. Liu Y. Zhu C. (2025). Dynamic kinetic resolution and dynamic kinetic asymmetric transformation of atropisomeric biaryls. Chem. Catal. 5:101329. doi: 10.1016/j.checat.2025.101329

[ref76] Zhang Q. Jiang L. Niu Y. Li Y. Chen W. Cheng J. . (2025). Machine learning-guided evolution of Pyrrolysyl-TRNA synthetase for improved incorporation efficiency of diverse noncanonical amino acids. Nat. Commun. 16, 6648–6648. doi: 10.1038/s41467-025-61952-2, 40681550 PMC12274524

[ref77] Zhang J. Jin S. Yun Q. Qu X. (2024). Biosynthesis of the unnatural extender units with polyketides and their structural modifications for applications in medicines. Synthetic Biol. J. 3, 561–570. doi: 10.12211/2096-8280.2023-093

[ref78] Zhang P. Liu C. Dai M. Wang G. Huang Y. Zhang L. . (2025). A one-pot multicomponent tandem reaction for the rapid synthesis of 2-Amino-3-Benzylindoles. Org. Biomol. Chem. 23, 3393–3399. doi: 10.1039/d5ob00187k, 40067208

[ref79] Zhang F. Qu G. Sun Z. Ma J. (2021). From chemical synthesis to biosynthesis: trends toward total synthesis of natural products. Synth. Biol. J. 5, 674–696. doi: 10.12211/2096-8280.2021-039

[ref80] Zhang Y. Szostak M. (2022). Synthesis of natural products by C−H functionalization of Heterocycless. Chem. Eur. J. 28:e202104278. doi: 10.1002/chem.202104278, 35089624 PMC9035081

[ref81] Zhang Q. Wu Y. Gong M. Zhang H. Liu Y. Lv X. . (2021). Production of proteins and commodity chemicals using engineered *Bacillus subtilis* platform strain. Essays Biochem. 65, 173–185. doi: 10.1042/ebc20210011, 34028523

[ref82] Zhang Y. Xu L. Xu Y. Sun Y. Hu X. (2024). Construction of a chassis cell with conditional suicide from *Pseudomonas putida* F1. Microbiology China 51, 5026–5036. doi: 10.13344/j.microbiol.china.240333

[ref83] Zhang Y. Zong J. Liu Y. Zhou K. Shi H. Yin W.-B. . (2025). Progress on targeted discovery of microbial natural products based on the predictions of both structure and activity. Nat. Prod. Rep. 42, 1651–1663. doi: 10.1039/d5np00008d 40625179

[ref84] Zhao L. He S. (2025). Research progress in total synthesis of daphenylline. Chem. Reagents 47, 1–12. doi: 10.13822/j.cnki.hxsj.2024.0462

[ref85] Zhou A. Amer M. M. K. Yin Q. (2026). Recent advances on asymmetric reduction via dynamic kinetic resolution. Chin. Chem. Lett. 37:111929. doi: 10.1016/j.cclet.2025.111929, 38826717

[ref86] Zhu M. Dai X. (2025). Systematic modulation of bacterial resource allocation by perturbing RNA polymerase availability via synthetic transcriptional switches. Nucleic Acids Res. 53:gkaf814. doi: 10.1093/nar/gkaf814, 40842238 PMC12370630

